# Oleic Acid Dissolves cGAS–DNA Phase Separation to Inhibit Immune Surveillance

**DOI:** 10.1002/advs.202206820

**Published:** 2023-03-22

**Authors:** Lina Wang, Qiaoling Liu, Na Wang, Siru Li, Wei Bian, Zhen Sun, Lulu Wang, Li Wang, Caigang Liu, Chengli Song, Quentin Liu, Qingkai Yang

**Affiliations:** ^1^ Institute of Cancer Stem Cell DaLian Medical University Dalian 116 044 P. R. China; ^2^ School of Life Science and Biotechnology Dalian University of Technology Dalian 116 024 P. R. China; ^3^ CAS Key Laboratory of Separation Sciences for Analytical Chemistry Dalian Institute of Chemical Physics Chinese Academy of Sciences Dalian 116 023 P. R. China; ^4^ Department of Oncology Shengjing Hospital of China Medical University Shenyang 110 004 P. R. China; ^5^ Sun Yat‐sen University Cancer Center State Key Laboratory of Oncology in South China Guangzhou 510 060 P. R. China

**Keywords:** cancer, cGAS, DNA‐sensing, phase separation

## Abstract

Phase separation (PS) is a fundamental principle in diverse life processes including immunosurveillance. Despite numerous studies on PS, little is known about its dissolution. Here, it is shown that oleic acid (OA) dissolves the cyclic GMP–AMP synthase (cGAS)–deoxyribonucleic acid (DNA) PS and inhibits immune surveillance of DNA. As solvent components control PS and metabolites are abundant cellular components, it is speculated that some metabolite(s) may dissolve PS. Metabolite‐screening reveals that the cGAS–DNA condensates formed via PS are markedly dissolved by long‐chain fatty acids, including OA. OA revokes intracellular cGAS–PS and DNA‐induced activation. OA attenuates cGAS‐mediated antiviral and anticancer immunosurveillance. These results link metabolism and immunity by dissolving PS, which may be targeted for therapeutic interventions.

## Graphical abstract

Phase separation (PS) is a fundamental principle for diverse life processes including immunosurveillance. Despite numerous studies on PS, little is known about the dissolution of PS. Oleic acid dissolves cGAS–DNA phase separation to inhibit immune surveillance, linking metabolism, and immunity via dissolving PS.

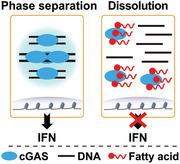

## 1 Introduction

Phase separation (PS) is crucial for life processes including immunosurveillance.^[^ ^1^ ^]^ To effectively mount immunoresponses and diminish harmful autoimmunity, the activation of immunosurveillance should be effectively and efficiently tuned.^[^ ^2^ ^]^ PS is potent in controlling molecule–molecule interactions and molecular concentrations.^[^ ^1^ ^]^ The critical roles of PS have been shown in the context of T cell receptor,^[^ ^3^ ^]^ B cell receptor,^[^ ^4^ ^]^ and cyclic guanosine monophosphate‐adenosine monophosphate synthase (cGAS).^[^ ^1^, ^5^ ^]^ However, little is known about the dissolution of sensor condensates formed via PS.

PS is a physicochemical phenomenon that is controlled by solvent components.^[^ ^1b^ ^]^ Notably, metabolites are abundant cellular components, and their levels are generally regulated by diet and metabolism.^[^ ^6^ ^]^ High fat diet (HFD) and metabolic syndromes, such as obesity, influence metabolite levels.^[^ ^7^ ^]^ Related studies show that they deregulate immunity thus increasing disease morbidities and mortalities.^[^ ^7^, ^8^ ^]^ Approximately 10% and 5% of cancers in women and men, respectively, are attributed to excess body weight.^[^ ^9^ ^]^ Owing to the role of PS in immunity,^[^ ^1^, ^5^ ^]^ HFD and obesity might deregulate certain metabolite(s) to dissolve the PS of immune protein (such as cGAS) to regulate immunity.

**Figure 1:**
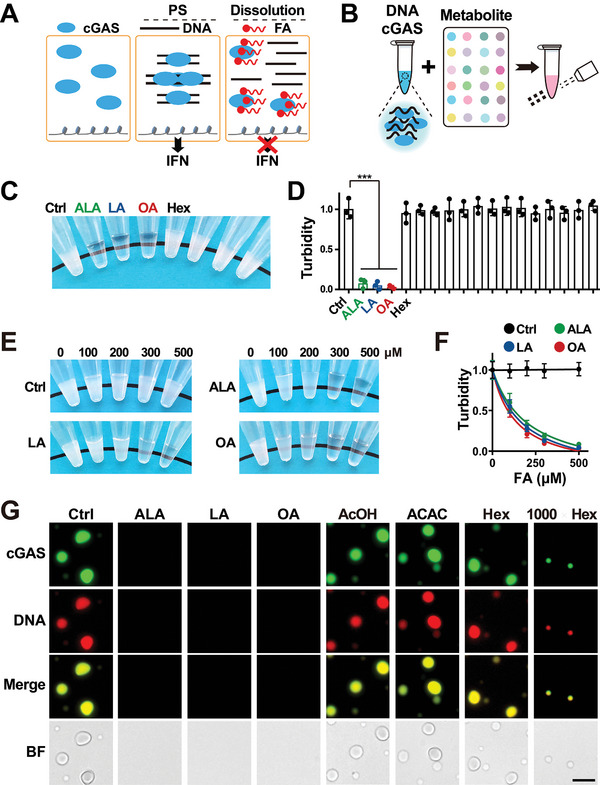
Metabolite screening revealed that FAs dissolve the cGAS–DNA phase separation. A) Schematic showing that fatty acids (FAs) dissolve the cellular cGAS–DNA phase separation (PS) to inhibit immunosurveillance against DNA. Every cGAS molecule needs to bind two condensed dsDNA strands (rather than one isolated dsDNA) to undergo cGAS–DNA PS to concentrate cGAS and DNA to activate cGAS. However, FA dissolves cGAS–DNA PS to inhibit surveillance of DNA. B) Schematic of metabolite screening. C) Representative images of the dissolution of cGAS–DNA PS. 20 µm of cGAS protein was mixed with 20 µm of 45‐bp oligo immune stimulatory DNA (ISD) in a buffer containing 20 mm of Tris‐HCl (power of hydrogen (pH) 7.5) and 150 mm of NaCl at 37 °C for 15 min. Then, the resultant mixtures containing cGAS–DNA condensates were incubated with 500 µm of the noted metabolite for 15 min. Unless specifically noted, distilled water was used as control (Ctrl) in this study. ALA: *α*‐linolenic acid; LA: linoleic acid; OA: oleic acid; Hex: 1,6‐hexanediol. D) Turbidities of the metabolite mixtures described in (C) were analyzed at 340 nm. The turbidity of Ctrl (distilled water) was considered to be 1. Unless specifically noted, Mean ± standard deviation (Mean ± SD) was used in this study. Unless specifically noted, *p* values were calculated by unpaired two‐tailed Student's *t*‐test, and *p* values < 0.05 were considered statistically significant (*p < 0.05, **p < 0.01, ***p < 0.001). *n* = 3. More details about the turbidities are shown in Table S1 of the Supporting Information. E) Representative images of the PS dissolution by the noted FAs at diverse concentrations. 20 µm of cGAS protein was mixed with 20 µm of ISD for 15 min. Then, the mixtures were incubated with the noted concentration of FAs for 15 min. F) Turbidities of the mixtures described in (E) were measured at 340 nm, and turbidity curves were built by plotting turbidities versus FA concentrations. *n* = 3. G) Representative fluorescent images of the dissolution of cGAS–DNA condensates by the noted metabolites. 20 µm of Alexa Fluor 488 (AF488)‐cGAS were mixed with 20 µm of Cy3‐ISD at 37 °C for 15 min. Then, the mixtures were incubated with 500 µm of metabolite for 15 min. Green: AF488‐labeled cGAS; Red: Cy3‐labeled ISD DNA; BF: bright field. AcOH: acetic acid; ACAC: acetoacetic acid; HEX: 500 µm of 1,6‐hexanediol; 1000× Hex: 0.5 m of 1,6‐hexanediol. Scale bar = 10 µm.

PS is pivotal for activation of cGAS, a central immune sensor (**Figure** **1**A).^[^ ^5a^ ^]^ cGAS binds deoxyribonucleic acid (DNA) to form cGAS–DNA PS to activate cGAS.^[^ ^5^, ^10^ ^]^ Activated cGAS synthesizes cyclic GMP–AMP (cGAMP) to mount immunoresponses, including interferon (Ifn) production.^[^ ^5^, ^10^ ^]^ The unique structure of the cGAS–DNA complex raises a central role for PS in the activation of cGAS.^[^ ^10^, ^11^ ^]^ Unlike other DNA sensors, every cGAS molecule needs to bind two condensed double‐stranded DNA strands (rather than one isolated double‐stranded DNA) to form the cGAS2n–DNA2 (n ≥ 1) complex to activate cGAS.^[^ ^10^, ^11^ ^]^ DNA molecules are negatively charged; therefore DNA condensation is energetically unfavorable.^[^ ^12^ ^]^ Moreover, the concentration threshold must be bypassed to activate cGAS.^[^ ^5^, ^11^, ^13^ ^]^ Notably, PS is well‐documented to concentrate molecule(s) to bypass the threshold of concentration.^[^ ^5^, ^10^, ^11^ ^]^ Therefore, PS is pivotal for the cGAS activation.^[^ ^5a^ ^]^ Interestingly, cGAS is crucial for both anti‐infection and anticancer immunity,^[^ ^5^, ^13^, ^14^ ^]^ which is attenuated by metabolic syndromes such as obesity.^[^ ^7^, ^8^ ^]^ These observations suggest a possibility for certain metabolite(s) (particularly obesity‐related metabolites) to dissolve the cGAS–DNA PS to attenuate surveillance.

Fatty acids (FAs) are metabolites related to obesity.^[^ ^7^, ^15^ ^]^ Oleic acid (OA) is the most abundant free FA (FFA) in sera.^[^ ^16^ ^]^ Long‐chain FAs contain a charged carboxylate hydrophilic end and a hydrocarbon hydrophobic end, which enables them to disperse one liquid into another immiscible liquid to dissolve the condensates formed via PS.^[^ ^17^ ^]^ Consistently, soaps (salts of long‐chain FAs) have been extensively used for centuries,^[^ ^17^ ^]^ raising the possibility for long‐chain FAs to dissolve intracellular PS. Long‐chain FA (hereafter referred to as FA) salt can potentially disrupt PS. FAs are controlled by diet and metabolism.^[^ ^7a,b^ ^]^ Moreover, HFDs and metabolic syndromes elevate FFA levels.^[^ ^7^ ^]^

In this study, we show that OA dissolves cGAS–DNA PS to revoke immunosurveillance against DNA (Figure 1A). OA inhibits cGAS‐binding, ‐condensation, and ‐activation by DNA. OA also reduces cGAS‐mediated antiviral and anticancer surveillance. Our study revealed a link between metabolism and immunity via PS dissolution, which may be targeted for therapeutic interventions.

## 2 Results

### 2.1 Metabolite Screening Revealed That FAs Dissolve the cGAS–DNA Phase Separation

PS, a physicochemical phenomenon, is physically controlled by solvent components,^[^ ^1b^ ^]^ and metabolites are the abundant cellular components.^[^ ^6^ ^]^ Interestingly, HFDs and metabolic syndromes such as obesity deregulate immunity,^[^ ^7^, ^8^ ^]^ which is controlled by PS.^[^ ^1^, ^5^, ^13^, ^14^ ^]^ These observations suggested that certain metabolites (particularly obesity‐related metabolites) might dissolve the PS such as the cGAS–DNA PS to attenuate immunity.

We screened a collection of obesity‐related metabolites^[^ ^18^ ^]^ by analyzing the turbidity of the cGAS–DNA condensates formed via PS^[^ ^19^ ^]^ (Figure 1B). The full‐length human cGAS protein was expressed and purified in vitro (Figure S1A, Supporting Information). To identify a potential metabolite for dissolving the cGAS–DNA PS, cGAS protein was mixed with oligo‐immune stimulatory DNA (ISD). As described in a previous study,^[^ ^5a^ ^]^ a mixture of cGAS protein and ISD led to a notable cGAS–DNA PS. The metabolite was then added to the mixture using distilled water as a control (Ctrl). After incubation, the turbidity of the resultant mixtures was assessed (Table S1, Supporting Information). Among the metabolites, FAs (particularly OA) markedly dissolved cGAS–DNA PS (Figure 1C,D; Table S1, Supporting Information). cGAS binds DNA to form condensates via ionic interaction,^[^ ^5^, ^10^ ^]^ and 1,6‐hexanediol ineffectively destroys ionic interaction‐mediated PS.^[^ ^1^, ^20^ ^]^ Consistently, 1,6‐hexanediol (Hex) poorly dissolved the cGAS–DNA condensates (Figure 1C,D). To examine the impacts of FA concentrations, serial dilutions of FAs were added to the mixtures containing cGAS–DNA condensates. As shown in Figure 1E,F, FA dissolved cGAS–DNA condensates in a concentration‐dependent manner.

To evaluate the impact of FAs on PS, fluorescence analyses were performed. Alexa Fluor 488 (AF488)‐labeled cGAS protein was mixed with Cy3‐labeled DNA. As previously described,^[^ ^5a^ ^]^ fluorescence recovery after photobleaching experiments showed that the fluorescence of cGAS or DNA in the condensates could be efficiently recovered (Figure S1B,C, Supporting Information). Therefore, the cGAS and DNA molecules in condensates may be mobile and exhibit dynamic internal rearrangement. In line with the turbidity experiment results, fluorescent analyses indicated that FAs markedly dissolved the cGAS–DNA PS, whereas 1,6‐hexanediol (Hex) poorly dissolved the PS (Figure 1G). Interestingly, short‐chain FAs, such as acetic acid (AcOH) and acetoacetic acid (ACAC), had little potential to dissolve the condensates, suggesting that the FA hydrophobic ends played a role in PS dissolution (Figure 1G). In line with the potential of FA to dissolve cGAS–DNA condensates, the simultaneous mixing of FA with cGAS and DNA revoked cGAS–DNA PS (Figure S1D, Supporting Information). Turbidity and fluorescence analyses showed that FAs dissolved PS in a time‐dependent manner (Figure S1E,F, Supporting Information), suggesting that FAs might bind cGAS or DNA to dissolve PS, but not robustly destroy the water shell of condensates to dissolve PS.

Additionally, we examined the impact of FAs on mouse cGAS–DNA PS. Full‐length mouse cGAS (mcGAS) protein was expressed and purified in vitro (Figure S1G, Supporting Information). Consistent with the results of human cGAS, turbidity experiments showed that FAs dissolved mouse cGAS–DNA PS in a concentration‐dependent manner (Figure S1H, Supporting Information). Similarly, fluorescence analyses indicated that FAs dissolved the mouse cGAS–DNA PS, while 1,6‐hexanediol (Hex) poorly dissolved the cGAS–DNA PS (Figure S1I,J, Supporting Information).

These results indicated that FAs dissolve cGAS–DNA PS.

### 2.2 FAs Inhibit the cGAS Binding and Activation by DNA In Vitro

**Figure 2:**
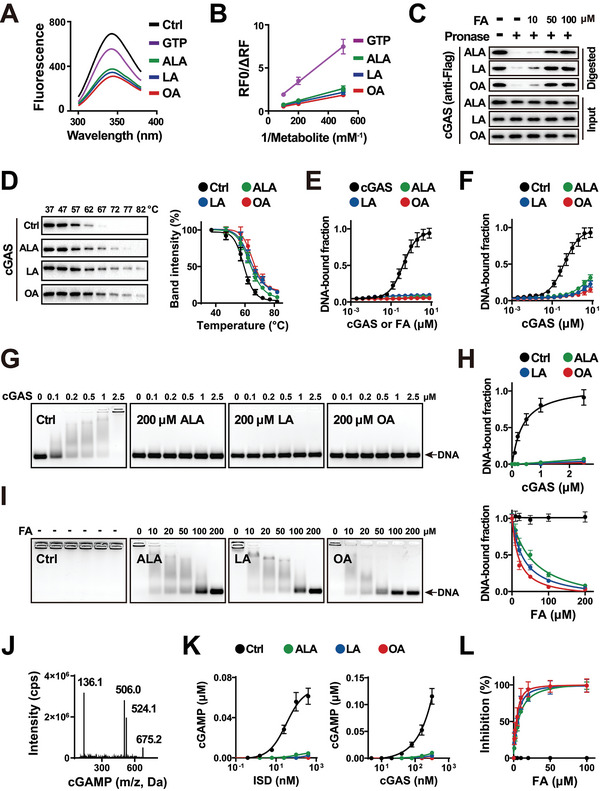
FAs inhibit the cGAS binding and activation by DNA in vitro. A) Metabolite affinity responsive target fluorescence quenching (MARTFQ) analyses of the quenching of cGAS intrinsic fluorescence by metabolite. 50 nm cGAS protein was mixed with 5 µm noted metabolite for 5 min, followed by the analyses of cGAS fluorescence. ALA: *α*‐linolenic acid; LA: linoleic acid; OA: oleic acid; GTP: guanosine triphosphate. B) Modified Stern–Volmer (RF0/ΔRF) curves to estimate the metabolite binding constants in (A). The binding constant (K value) was estimated by the modified Stern–Volmer equation: RF0/ΔRF = (1/*fK*) × (1/[*Q*]) + (1/*f*). Mean ± SD. *n* = 3. C) Drug affinity responsive target stability (DARTS) analyses of cGAS binding to the noted FAs. MEF cells were transfected with pCDH Flag cGAS‐FL plasmid for 36 h. Then, the cell lysates were incubated with the noted FA for 1 h and then Pronase (a protease) for 30 min, followed by immunoblotting analyses with anti‐Flag antibody. Input: the cell lysates treated without Pronase; Digested: the cell lysates treated with Pronase. FA: fatty acid; ALA: *α*‐linolenic acid; LA: linoleic acid; OA: oleic acid. D) Cellular thermal shift assays (CETSAs) of cGAS binding to the noted FAs. MEF cells were treated with 1 mm noted FAs (FA: fatty‐acid‐free BSA = 5: 1) for 2 h, and then heated at the noted temperatures for 3 min. Left: immunoblotting of the cell lysates using anti‐cGAS antibody. Right: immunoblotting band intensity versus temperature. Ctrl: distilled water; ALA: *α*‐linolenic acid; LA: linoleic acid; OA: oleic acid. *n* = 3. E) Microscale thermophoresis (MST) assays of DNA binding to cGAS or FAs. 50 nm of Cy5‐labeled ISD was mixed with serial dilutions of cGAS protein or the noted FA. Then, the thermophoretic movement of DNA was analyzed by Monollith instrument. *n* = 3. F) MST assays of DNA binding to cGAS in the noted FAs. 50 nm of Cy5‐labeled ISD and 100 of µm noted FA were mixed with a serial dilution of the cGAS protein. *n* = 3. G) Electrophoretic mobility shift assays (EMSAs) of cGAS binding to ISD in 200 µm FAs. 200 µm FA and 50 nm ISD were mixed with serial dilutions of cGAS protein for 30 min. Ctrl: distilled water; ALA: *α*‐linolenic acid; LA: linoleic acid; OA: oleic acid. H) DNA bound fraction of EMSA analyses described in (G). *n* = 3. I) EMSA analyses of cGAS protein binding to ISD in serial dilutions of noted FAs. 50 nm ISD and 2.5 µm cGAS were incubated with serial dilutions of FAs for 30 min. Ctrl: distilled water; FA: fatty acid; ALA: *α*‐linolenic acid; LA: linoleic acid; OA: oleic acid. J) Ion mass transition of 2′3′‐cGAMP in MS analyses. cps: counter per second. K) Left: MS analyses of the cGAMP produced by the mixing of 50 µm noted FA, 200 nm cGAS, and a serial dilution of ISD for 30 min. Right: cGAMP produced by mixing 50 µm noted FA, 200 nm ISD, and a serial dilution of cGAS for 30 min. *n* = 3. L) Serial dilutions of FA inhibited the cGAMP production in the mixture of 200 nm of cGAS and 200 nm of ISD for 30 min. FA: fatty acid. *n* = 3.

Molecule–molecule interaction is central to PS.^[^ ^1b^ ^]^ This was emphasized by the observation that FAs dissolved PS in a time‐dependent manner, suggesting that FAs might bind cGAS or DNA to dissolve PS, but not robustly destroy the water shell of condensates formed via PS. Therefore, we examined the interaction between cGAS and FAs. Based on metabolite‐induced intrinsic fluorescence quenching of the target protein, metabolite affinity responsive target fluorescence quenching (MARTFQ) analyses revealed that FAs were more potent in quenching the cGAS intrinsic fluorescence than GTP (a substrate of cGAS as a positive control) (**Figure** **2**A,B; Figure S2A, Supporting Information), thus indicating that FAs could bind cGAS. Similar results were observed in drug affinity‐responsive target stability (DARTS) assays. The DARTS assay is based on a reduction in protease susceptibility of the target protein upon ligand binding. In the presence or absence of FAs, Pronase (a protease) was used to digest cGAS proteins. As shown in Figure 2C, DARTS assays demonstrated that FAs reduced the protease susceptibility of cGAS, indicating that FAs bound to cGAS. Cellular thermal shift assays (CETSAs) were performed to examine the potentials of FAs to bind to intracellular cGAS. CETSA takes advantage of ligand‐enhanced thermal stabilization of intracellular target protein. Mouse embryonic fibroblast (MEF) cells were treated with FAs and then heated at various temperatures. The resultant cell lysates were subjected to immunoblotting with an anti‐cGAS antibody. Consistent with the MARTFQ and DARTS results, CETSA showed that FAs enhanced the thermal stabilization of cGAS, indicating that FAs bound cGAS (Figure 2D). Additionally, circular dichroism (CD) analysis was used to evaluate the impact of FAs on the conformation of cGAS. As shown in Figure S2B of the Supporting Information, FAs had little impact on the cGAS conformation. This observation was consistent with studies showing that metabolites, even as substrates of target proteins, might show little potential to change protein conformation.^[^ ^21^ ^]^ For example, 2‐oxoglutarate is a substrate of DNA oxidative demethylase^[^ ^21a^ ^]^ and aurine dioxygenase,^[^ ^21b^ ^]^ but 2‐oxoglutarate has little potential to change the conformation of DNA oxidative demethylase and aurine dioxygenase.^[^ ^21^ ^]^

We evaluated the interaction between DNA and FAs using microscale thermophoresis (MST). The MST assay detects changes in the hydration shells of molecules and measures biomolecule interactions under close‐to‐native conditions. cGAS protein or FA was mixed with fluorescence‐labeled DNA, followed by MST analysis of DNA thermophoretic movement. As shown in Figure 2E, cGAS bound DNA markedly, while FAs bound DNA poorly. These results suggest that FAs might bind cGAS to dissolve cGAS–DNA PS.

Next, we examined the impact of FAs on cGAS–DNA binding using MST and electrophoretic mobility shift assay (EMSA). For MST assays, cGAS protein and FA were simultaneously mixed with DNA, using drilled water as a control (Ctrl). As shown in Figure 2F, FAs notably reduced cGAS–DNA binding. Based on the principle that the electrophoretic mobility of a protein–DNA complex is less than that of free DNA, EMSA was used to assess the impact of FAs on cGAS–DNA binding. In the context of FAs at constant concentrations, EMSA showed that FAs elevated the electrophoretic mobility of DNA, suggesting that FAs reduced the cGAS–DNA binding (Figure 2G,H). In the context of cGAS at constant concentration, EMSA indicated that FAs decreased cGAS–DNA binding in a concentration‐dependent manner (Figure 2I).

Furthermore, we evaluated the role of FAs in DNA‐induced cGAS activation. Mass spectrometry (MS) analyses were performed to measure the cGAMP produced by cGAS (Figure 2J). Considering that both cGAS and DNA concentrations influence cGAS activity,^[^ ^5^, ^11^ ^]^ constant concentration of cGAS or DNA was used for cGAS activity assays. In line with the impact of FA on cGAS–DNA binding, FAs blocked the cGAS activation by DNA (Figure 2K). FA inhibited cGAS activity in a dose‐dependent manner (Figure 2L). Additionally, the impact of FAs on the kinetic parameters of cGAS was assessed. As shown in Figure S2C–F of the Supporting Information, FAs effectively decreased *K*_cat_ and increased *K*_m_, thus inhibiting cGAS activity (*K*_cat_/*K*_m_).

Additionally, we evaluated the potentials of FAs to inhibit the mouse cGAS (mcGAS) binding and activation by DNA. Consistent with the results of human cGAS, EMSA experiments showed that FAs elevated the electrophoretic mobility of DNA in a dose‐dependent manner, suggesting that OA reduced mouse cGAS–DNA binding (Figure S2G,H, Supporting Information). We assessed the effect of FAs on mouse cGAS activation by DNA. Consistent with the effect of FAs on mouse cGAS–DNA binding, FAs blocked the mouse cGAS activation by DNA (Figure S2I, Supporting Information). FAs inhibited mouse cGAS activity in a dose‐dependent manner (Figure S2J, Supporting Information).

Collectively, the above results indicated that FAs inhibited cGAS‐binding and ‐activation by DNA. Due to the potential of FA to bind cGAS, FA might bind cGAS to inhibit the cGAS‐binding and ‐activation by DNA.

### 2.3 cGAS–OA Binding Might Be Regulated by power of hydrogen

**Figure 3:**
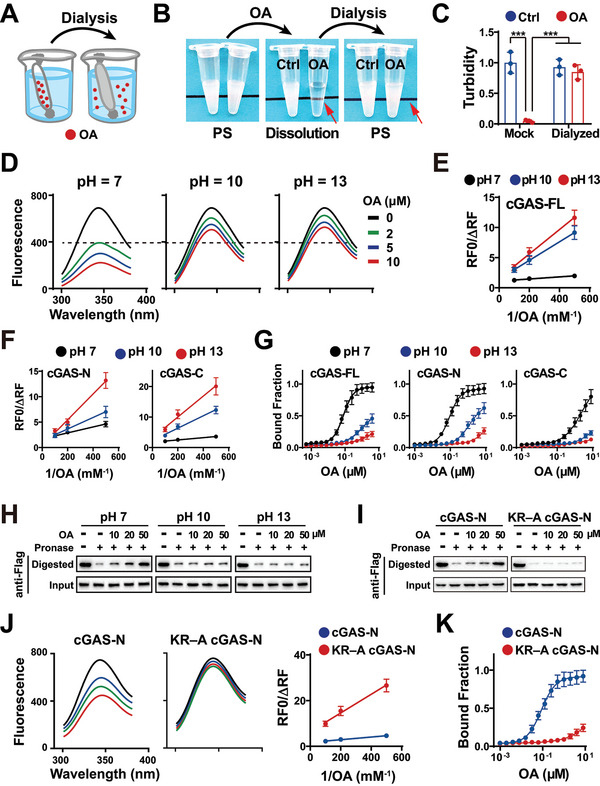
cGAS–OA binding might be regulated by pH. A) Schematic showing that dialysis removes OA from the cGAS–DNA–OA mixture to recover the cGAS–DNA PS. OA: oleic acid. B) Representative images of the recovery of the cGAS–DNA PS via dialysis. 20 µm cGAS was mixed with 20 µm HT‐DNA for 15 min. Then, 500 µm OA was added to the mixture to dissolve the condensates using distilled water as control (Ctrl). Resultant mixtures were dialyzed using cellulose dialyzer membrane (molecular weight cut‐off: 5000 Da) to remove OA and recover the condensation. C) Turbidities of the mixtures described in (B). Unless specifically noted, mean ± standard deviation (mean ± SD) was used in this study. *P* values were calculated by unpaired two‐tailed Student's *t*‐test, and *p* values < 0.05 were considered statistically significant (*p < 0.05, **p < 0.01, ***p < 0.001). *n* = 3. D) MARTFQ analyses of the quenching of cGAS fluorescence by a serial dilution of OA at the distinct pH. 50 nm of full‐length cGAS protein was mixed with OA at the noted power of hydrogen (pH) for 5 min. Then, the samples were subjected to fluorescence analyses. E) Modified Stern–Volmer (RF0/ΔRF) curves to estimate the binding constants between OA and full‐length cGAS (cGAS‐FL) at the noted pH. *n* = 3. F) Modified Stern–Volmer (RF0/ΔRF) curves for OA and cGAS truncated proteins at the noted pH. A serial dilution of OA was mixed with 100 nm of cGAS‐N or ‐C protein at the noted pH for 5 min. cGAS‐N: N‐terminal cGAS truncated protein; cGAS‐C: C‐terminal cGAS truncated protein. *n* = 3. G) MST assays of cGAS proteins binding to OA at the noted pH. 100 nm Cy5‐labeled cGAS proteins were mixed with a serial dilution of OA at the noted pH. Then, the thermophoretic movements of proteins were analyzed. *n* = 3. H) DARTS analyses of cGAS binding to OA at the noted pH. MEF cells were transfected with pCDH Flag cGAS‐FL plasmid for 36 h. Then, the cell lysates were mixed with OA at the noted pH for 1 h. After incubation with Pronase for 30 min, the resultant mixtures were subjected to immunoblotting with the anti‐Flag antibody. Input: the cell lysates treated without Pronase; Digested: the cell lysates treated with Pronase. OA: oleic acid. *n* = 3. I) DARTS analyses of KR–A cGAS‐N protein binding to OA. MEF cells were transfected with pCDH Flag cGAS‐N or pCDH Flag KR–A cGAS‐N plasmid. Then, the cell lysates were mixed with a serial dilution of OA at pH 7. Following incubation with Pronase, the resultant mixtures were subjected to immunoblotting with the anti‐Flag antibody. Input: the cell lysates treated without Pronase; Digested: the cell lysates treated with Pronase. OA: oleic acid. *n* = 3. J) MARTFQ analyses of the quenching of cGAS‐N and KR–A cGAS‐N protein fluorescence by a serial dilution of OA. 50 nm of noted protein was mixed with OA at pH 7 for 5 min, followed by fluorescence analyses. *n* = 3. K) MST assays of cGAS‐N and KR–A cGAS‐N proteins binding to OA. 100 nm Cy5‐labeled cGAS‐N or KR–A cGAS‐N protein was mixed with a serial dilution of OA at pH 7. Then, the thermophoretic movements of proteins were analyzed. *n* = 3.

To investigate the mechanism by which FAs bind to cGAS, we first evaluated the reversibility of cGAS–OA binding via dialysis (**Figure** **3**A). Dialysis experiments indicated that the removal of OA restored cGAS–DNA PS (Figure 3B,C), suggesting that FAs bind cGAS in a reversible manner.

Next, we assessed the thermodynamic parameters of cGAS–OA binding via fluorescence polarization (FP) and isothermal titration calorimetry (ITC). Fluorescently labeled molecules rotate rapidly between excitation and emission, leading to the emission of mostly depolarized light. Fluorescent molecule–protein binding reduces the rotation of fluorescent molecules, resulting in the emission of largely polarized light. Based on this phenomenon, FP was used to assess the interaction between cGAS and BODIPY‐labeled OA. As shown in Figure S3A of the Supporting Information, FP analyses demonstrated that cGAS increased the polarization of OA‐emitted fluorescence, suggesting that OA bound to cGAS. ITC titration of OA to the cGAS protein was performed. ITC analysis is based on the principle that the interaction between two molecules results in heat generation or absorption. ITC titration assays demonstrated that the free energy (Δ*G*) of cGAS–OA binding was <0, indicating that cGAS–OA binding generates heat (Figure S3B, Supporting Information). The heat generation indicated that cGAS–OA binding is energetically favorable. Notably, the OA:cGAS molar ratio in ITC assays showed that one cGAS molecule bound to multiple OA molecules, suggesting that cGAS contains multiple OA‐binding sites.

To determine the domain of cGAS that binds FAs, N‐terminal and C‐terminal (catalytic domain) truncated cGAS proteins were expressed and purified (Figure S3C, Supporting Information) as previously described.^[^ ^5a^ ^]^ Consistent with the results of full‐length cGAS (cGAS‐FL), both N‐terminal cGAS (cGAS‐N) and C‐terminal cGAS (cGAS‐C, catalytic domain) could undergo PS with DNA, while OA effectively dissolved the condensates formed via PS (Figure S3D, Supporting Information). Furthermore, EMSA showed that OA inhibited the binding of cGAS‐N (Figure S3E, Supporting Information) and cGAS‐C (Figure S3F, Supporting Information) to DNA. These results suggest a shared mechanism by which OA binds cGAS‐FL, cGAS‐N, and cGAS‐C proteins.

Structural studies have shown that cGAS binds to DNA mainly via multiple basic lysine (K) and arginine (R) residues of cGAS^[^ ^10c^ ^]^ (Figure S3G, Supporting Information), which usually display positive charges at power of hydrogen (pH) ≈7. These observations suggested that a shared characteristic of cGAS‐FL, cGAS‐N, and cGAS‐C proteins might be their relatively high isoelectric point (pI) (Figure S3H, Supporting Information). In line with the impact of pH on electrostatic/ionic interactions, further analyses indicated that high pH reduced cGAS–DNA binding (Figure S3I, Supporting Information).

We then evaluated the impact of pH on cGAS–FA binding via MARTFQ, MST, and DARTS assays. MARTFQ analyses showed that high pH decreased the potential of FA to quench the intrinsic fluorescence of the cGAS‐FL protein (Figure 3D,E, Supporting Information) and truncated cGAS proteins (Figure 3F), indicating that high pH reduced cGAS–FA binding. Consistently, MST assays demonstrated that high pH attenuated the FA potential to reduce the thermophoretic movement of cGAS proteins (Figure 3G), indicating that high pH decreased cGAS–FA binding. The role of pH was assessed using DARTS. Consistent with the results of MARTFQ and MST, DARTS showed that high pH attenuated the FA potential to decrease protease susceptibility of cGAS proteins (Figure 3H; Figure S3J, Supporting Information).

To investigate the mechanism by which FAs bind to cGAS, we replaced the basic K and R residues with neutral alanine (A) residues to build neutral KR–A cGAS‐N mutant (Figure S3K,L, Supporting Information). As shown in Figure 3I, DARTS assays indicated that, at near neutral pH, OA considerably reduced the protease susceptibility of cGAS‐N to a greater extent than KR–A cGAS‐N. Therefore, the neutral KR–A mutation diminished the potential of OA to reduce the protease susceptibility of cGAS, indicating that basic K and R residues play a role in OA–cGAS binding. Similarly, MARTFQ analyses indicated that OA effectively quenched cGAS‐N fluorescence but not KR–A cGAS‐N fluorescence at near neutral pH (Figure 3J), raising the role of these basic residues in OA–cGAS binding. Consistently, MST analyses also demonstrated that the KR–A mutation reduced the potential of cGAS to bind to OA at near‐neutral pH (Figure 3K).

These results suggested that cGAS–OA binding is regulated by pH.

### 2.4 FA Dissolves the Cellular cGAS–DNA Condensates Formed via Phase Separation

**Figure 4:**
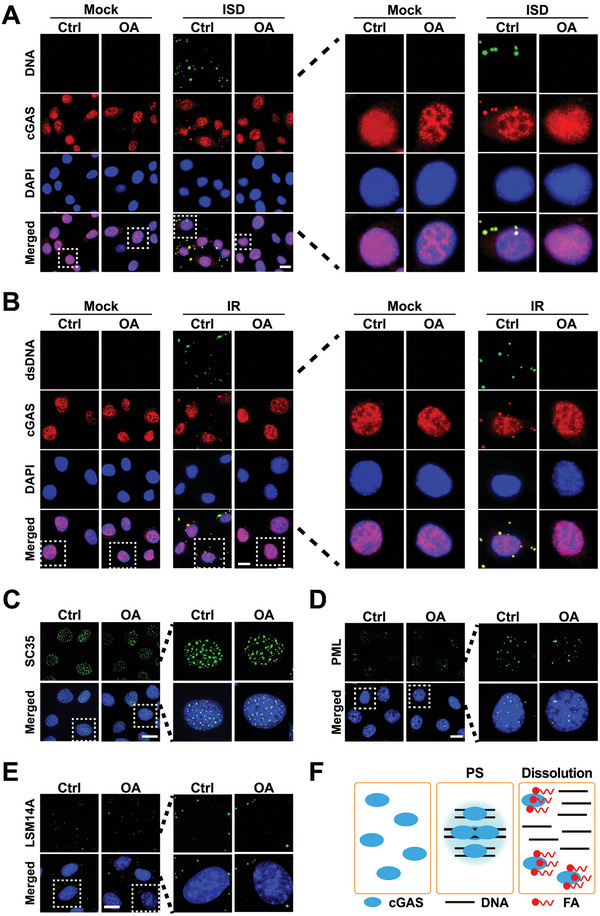
FA dissolves the intracellular cGAS–DNA phase separation. A) Representative immunofluorescence (IF) images of the cGAS–DNA condensates in the cells treated with DNA transfection and then OA. Mouse embryonic fibroblast (MEF) cells were transfected with 1 µg mL^−1^ FAM‐labeled oligo immune stimulatory DNA (ISD) for 0.5 h. Then, the cells were treated with 1 mm of OA (OA:BSA = 5:1) for 4 h, and subjected to IF analyses with anti‐cGAS antibody. Unless specifically noted, distilled water was used as control (Ctrl). Red: anti‐cGAS antibody; Green: FAM‐labeled ISD DNA; Blue: DAPI. Scale bar = 10 µm. *n* = 3. B) Representative IF images of the cGAS–DNA condensates in the cells treated with IR and then OA. MEF cells were treated with 8 Gy of irradiation (IR). After 1 day, the cells were treated with 1 mm of OA for 4 h, and then subjected to IF analyses with anti‐cGAS and anti‐dsDNA antibodies as noted. Red: anti‐cGAS antibody; Green: anti‐dsDNA antibody; Blue: DAPI. Scale bar = 10 µm. *n* = 3. C) Representative IF images of the nuclear speckles in the cells treated with OA. MEF cells were treated with 1 mm of OA for 4 h, and then subjected to IF analyses with anti‐SC35 (serine and arginine rich splicing factor 2) antibody. Red: anti‐SC35 antibody; Blue: DAPI. Scale bar = 10 µm. *n* = 3. D,E) Representative IF images of the PML nuclear bodies (D) and processing bodies (E) in cells treated with OA. The MEF cells were treated with 1 mm OA for 4 h, and then subjected to IF analyses with anti‐PML nuclear body scaffold (PML) (D) or LSM14A mRNA processing body assembly factor (LSM14A) (E) antibody as noted. Green: anti‐PML (D) or anti‐LSM14A (E) antibodies; Blue: DAPI. Scale bar = 10 µm. *n* = 3. F) Schematic showing that cGAS binds DNA to undergo the cGAS–DNA PS, while FA dissolves the intracellular cGAS–DNA condensates formed via PS.

To determine whether FA dissolved the intracellular cGAS–DNA condensates formed via PS, the cells were treated with oleic acid (OA), the main FA component in serum FFAs.^[^ ^16^ ^]^ Immunofluorescence (IF) analyses were used to evaluate the cGAS–DNA PS in cells treated with OA before and after DNA transfection. As previously described,^[^ ^5a^ ^]^ the presence of transfected DNA induced the cytosolic mouse cGAS–DNA PS (**Figure** **4**A). In line with the in vitro results, OA treatment after DNA transfection led to the dissolution of mouse cGAS–DNA condensates formed via PS (Figure 4A), while OA treatment before DNA transfection inhibited the mouse cGAS–DNA PS (Figure S4A, Supporting Information). Because cytosolic DNA is a damage‐associated molecular pattern,^[^ ^13^ ^]^ we determined whether FA dissolved the irradiation (IR)‐promoted mouse cGAS–DNA PS. As previously described,^[^ ^22^ ^]^ IR induced cytosolic DNA accumulation and mouse cGAS–DNA PS (Figure 4B). In line with the DNA transfection results, OA treatment dissolved the IR‐promoted mouse cGAS–DNA condensates formed via PS (Figure 4B). Similarly, in human U2OS cells, OA dissolved the transfected DNA–cGAS condensates (Figure S4B, Supporting Information) and IR‐promoted DNA–cGAS condensates (Figure S4C, Supporting Information).

Subsequently, we evaluated the influence of OA on the well‐known cellular condensates formed via PS. IF analyses showed that OA treatment had little impact on nuclear speckles (Figure 4C), PML nuclear bodies (Figure 4D), processing bodies (Figure 4E), and arsenic acid (As)‐promoted stress granules (Figure S4D, Supporting Information), raising the impact of OA on cellular cGAS–DNA PS.

Additionally, we evaluated the effect of OA on human and mouse cellular cGAS–DNA binding using immunoprecipitation. Mouse and human cell lysates were mixed separately with biotin‐labeled ISD and/or OA. The mixtures were then incubated with streptavidin‐coupled Dynabead. Electrophoresis analyses of the resultant precipitates showed that DNA bound to both human and mouse cellular cGAS proteins, and OA abrogated the potential of DNA to bind to the cellular cGAS proteins (Figure S4E, Supporting Information).

These results indicate that FA can dissolve intracellular cGAS–DNA PS (Figure 4F).

### 2.5 FA Inhibits the Cellular cGAS Activation

**Figure 5:**
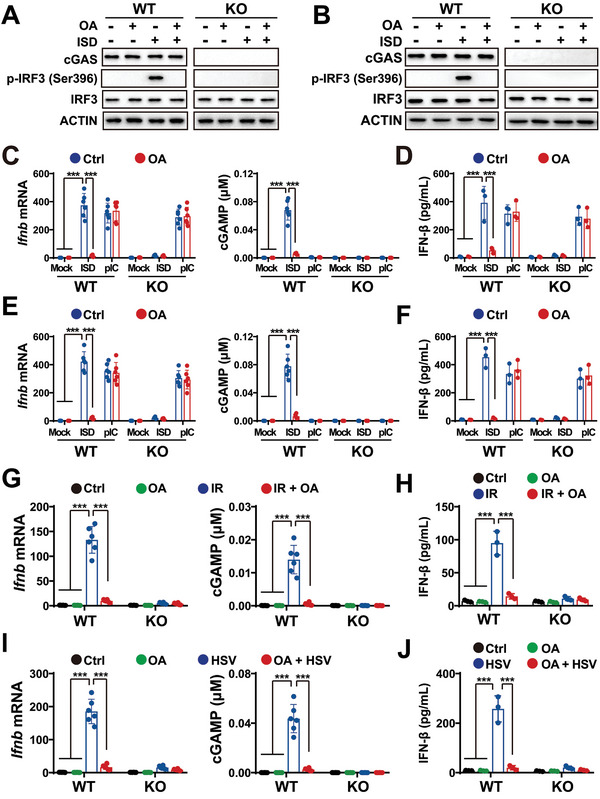
FA inhibits the cellular cGAS activation. A) Immunoblotting analyses of the lysates from cGAS wild type (WT) and knockout (KO) MEF cells treated with 1 µg mL^−1^ of ISD transfection for 0.5 h and then 1 mm of OA for 8 h. IRF3: interferon regulatory factor 3; p‐IRF3 (ser396): phospho‐interferon regulatory factor 3 (Ser396). WT: wild type; KO: knockout. ISD: oligo immune stimulatory DNA. OA: oleic acid. *n* = 3. B) Immunoblotting analyses of the lysates from the MEF cells treated with 1 mm of OA for 1 h and then 1 µg mL^−1^ of ISD transfection for 8 h. IRF3: interferon regulatory factor 3; p‐IRF3 (ser396): phospho‐interferon regulatory factor 3 (Ser396). WT: wild type; KO: knockout. ISD: oligo immune stimulatory DNA. OA: oleic acid. *n* = 3. C) Ifnb mRNA and cGAMP levels in the cells treated with transfection and then OA. cGAS WT and KO MEF cells were treated with 1 µg mL^−1^ of ISD or poly (I:C) (pIC) dsRNA transfection for 0.5 h, and then 1 mm of OA for 8 h. Ifnb mRNA and cGAMP levels in the resultant cells were analyzed by qRT‐PCR and MS, respectively. ISD: 45‐bp oligo immune stimulatory DNA. pIC: poly (I:C) dsRNA. Unless specifically noted, mean ± standard deviation (mean ± SD) was used in this study. Unless specifically noted, *p* values were calculated by unpaired two‐tailed Student's *t*‐test, and *p* values < 0.05 were considered statistically significant (*p < 0.05, **p < 0.01, ***p < 0.001). *n* = 6. D) Enzyme linked immunosorbent assays (ELISAs) of the secretion of IFN‐*β* protein from the cells treated with 1 µg mL^−1^ of ISD or pIC dsRNA transfection for 0.5 h, and then 1 mm of OA for 12 h. *n* = 3. E) Ifnb mRNA and cGAMP levels in the MEF cells treated with 1 mm of OA for 1 h, and then 1 µg mL^−1^ ISD or pIC dsRNA transfection for 8 h. *n* = 6. F) IFN‐*β* protein secreted from the cells treated with 1 mm of OA for 1 h, and then ISD or pIC dsRNA transfection for 12 h. *n* = 3. G) Ifnb mRNA and cGAMP levels in the MEF cells treated with irradiation (IR) and/or OA. cGAS WT and KO MEF cells were treated with 8 Gy of IR. After 1 h, the cells were treated with 1 mm of OA for 24 h. *n* = 6. H) IFN‐*β* protein secreted from the cells treated with IR and/or OA as described in (G). *n* = 3. I) Ifnb mRNA and cGAMP levels in the MEF cells treated with OA and/or herpes simplex virus‐1 (HSV) infection. cGAS WT and KO MEF cells were treated with 1 mm of OA for 1 h, and then five multiplicity of infection (MOI) of HSV infection for 8 h. *n* = 6. J) IFN‐*β* protein secreted from the cells treated with OA and/or HSV infection. The MEF cells were treated with 1 mm OA for 1 h, and then 5 MOI HSV infection for 12 h. *n* = 3.

Because of the role played by PS in cGAS activation,^[^ ^5a^ ^]^ we evaluated the impact of FA on the cGAS activation in MEF cells. As shown in **Figure** **5**A,B, OA treatments inhibited the phosphoactivation of interferon regulatory factor 3 (IRF3), suggesting that FA inhibited cellular cGAS activation by DNA. Both OA treatment and cGAS knockout (KO) blocked the IFN‐*β* and cGAMP productions, and showed poor impact on poly I:C dsRNA (pIC)‐induced IFN‐*β* production (Figure 5C–F), indicating that OA inhibited surveillance against DNA but not dsRNA. Notably, OA and cGAS KO showed little additive effects, suggesting that FA might regulate immune surveillance via cGAS (Figure 5C–F). To further evaluate the effect of OA, we examined the impact of OA on the expression of genes involved in multiple metabolism and immune pathways. Real‐time quantitative reverse transcription PCR (qRT‐PCR) analyses indicated that OA had little impact on the expression of genes involved in amino acid, glucose, glycogen, polyamine, and lipid metabolism pathways (Figure S5A, Supporting Information). Conversely, OA considerably decreased the DNA transfection‐elevated expression of cytokines, chemokines, and interferon‐stimulated genes (Figure S5A, Supporting Information), which are downstream genes of the cGAS pathway.^[^ ^22^, ^23^ ^]^ To a less extent, OA impaired the DNA transfection‐increased expression of nuclear factor‐kB pathway members (Figure S5A, Supporting Information), which are moderately regulated by cGAS pathway.^[^ ^24^ ^]^ These observations at least partially suggest that OA regulates cGAS activation. Subsequently, we assessed the effect of FA on IR‐induced cGAS activation. As shown in Figure 5G,H, OA inhibited IR‐induced cGAS activation. Consistently, qRT‐PCR analyses showed that OA decreased the IR‐elevated expression of the downstream genes of the cGAS pathway^[^ ^22^, ^23^ ^]^ but had little impact on the expression of the genes involved in metabolic pathways (Figure S5B, Supporting Information).

Considering that diet and metabolism can regulate immunity to control infection morbidity and mortality,^[^ ^8a,b^ ^]^ we examined the role of FA in cGAS activation by herpes simplex virus‐1 (HSV). As indicated by the results of DNA transfection, OA treatment and cGAS KO blocked HSV‐stimulated IFN‐*β* and cGAMP production, but not additively (Figure 5I,J), suggesting that OA inhibited HSV‐promoted cGAS activation. Moreover, qRT‐PCR analyses showed that OA decreased the HSV infection‐elevated expression of the downstream genes of the cGAS pathway,^[^ ^22^, ^23^ ^]^ but had little impact on the metabolism pathway genes (Figure S5C, Supporting Information).

Additionally, we evaluated the effect of OA on cGAS activation in human THP‐1 cells and found that it was similar to that observed in MEF cells (Figure S5D–K, Supporting Information). These results indicated that FA inhibited cellular cGAS activation.

### 2.6 FAs Reduce the cGAS‐Mediated Antivirus Immunity

**Figure 6:**
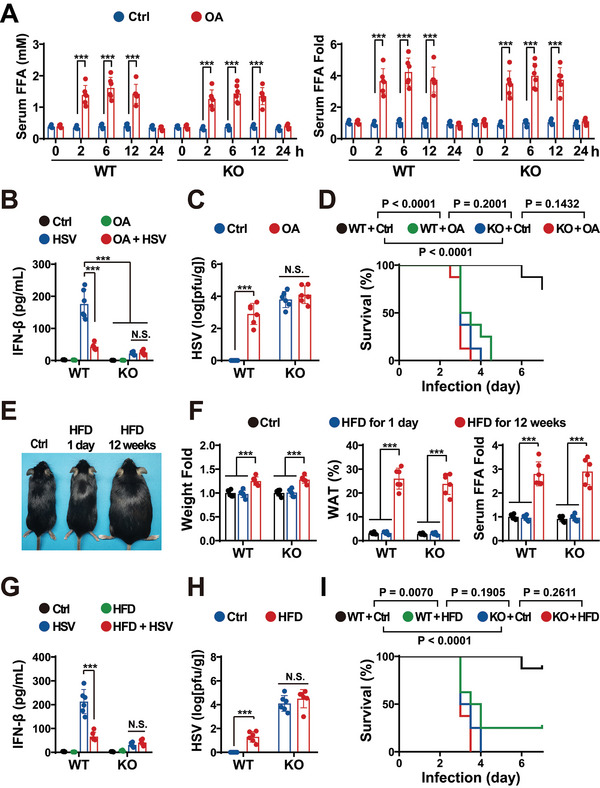
FAs reduce cGAS‐mediated antivirus immunity. A) Absolute (left) and relative (right) free fatty acid (FFA) levels in the sera from the mice intraperitoneally (IP) injected with 20 mg per mouse OA (OA/BSA of 5:1) using BSA as control (Ctrl). Unless specifically noted, mean ± standard deviation (mean ± SD) was used in this study. Unless specifically noted, *p* values were calculated by unpaired two‐tailed Student's *t*‐test, and *p* values < 0.05 were considered statistically significant (*p < 0.05, **p < 0.01, ***p < 0.001). *n* = 6 mice. B) ELISA analyses of the IFN‐*β* protein levels in the sera from the mice treated with OA injection and herpes simplex virus‐1 (HSV) infection. Mice were IP injected with 20 mg per mouse OA. After 2 h, the mice were intravenously (IV) infected by 1 × 10^7^ plaque forming units (pfu) HSV. The sera were collected at 4 h postinfection. N.S.: No significance, *p* > 0.05. *n* = 6 mice. C) The viral titer in the brains of the mice treated with OA and HSV. Mice were IP injected with OA at 20 mg per mouse. After 2 h, the mice were IV infected by 1 × 10^6^ pfu HSV. Then, the mice were IP injected with OA once a day for 3 days. The viral titers in the brain homogenates were assessed by the plaque assay. *n* = 6 mice. D) Kaplan–Meier (KM) analyses of the survival in the mice treated with OA and HSV infection. Mice were IP injected with OA at 20 mg per mouse. After 2 h, the mice were IV infected by 1 × 10^6^ pfu HSV. Then, the mice were treated with the IP injection of 20 mg per mouse OA once a day. The mouse survivals were monitored for 7 days. *P* values were obtained from the log‐rank test. *n* = 8 mice. E) Representative images of the mice fed with standard rodent chow (Ctrl) or high fat diet (HFD) (60 kcal% fat diet) ad libitum for the noted times. F) Body weight, white adipose tissue (WAT), and serum FFA levels in the mice described in (E). WAT was assessed as described in our previous study.^[^ ^18^ ^]^ *n* = 6 mice. G) ELISA analyses of the IFN‐*α* and IFN‐*β* protein levels in the sera from the mice treated with HFD and HSV. Mice pretreated with or without HFD for 12 weeks were IV infected by 1 × 10^7^ pfu HSV for 4 h. *n* = 6 mice. H) The viral titer in the brains from the mice treated with HFD and HSV. Mice pretreated with or without HFD for 12 weeks were infected with 1 × 10^6^ pfu HSV. For HFD groups, the mice were consistently treated with HFD during the experiment. After 3 days, the brain viral titers were assessed. *n* = 6 mice. I) Kaplan–Meier (KM) analyses of the survival in the mice treated with HFD and HSV. Mice pretreated with or without HFD for 12 weeks were infected with 1 × 10^6^ pfu HSV. The survivals were monitored for 7 days. *P* values were obtained from the log‐rank test. *n* = 8 mice.

As mentioned above, HFDs and metabolic syndromes attenuate immunity and increase the morbidity and mortality rates of infections.^[^ ^8a,b^ ^]^ To evaluate the role of FA in the cGAS‐mediated anti‐infection immunity, we treated cGAS wild type (WT) and knockout (KO) mice with OA injection and/or HSV infection. As shown in **Figure** **6**A, intraperitoneal (IP) injection of OA increased FFA levels. Consistent with the in vitro results, OA injection and cGAS KO separately reduced HSV‐stimulated IFN production, but no substantial additive effect was detected, suggesting that FA might inhibit IFN production via cGAS (Figure 6B; Figure S6A,B, Supporting Information). Consistently, qRT‐PCR analyses showed that OA decreased the HSV infection‐elevated expression of the downstream genes of the cGAS pathway^[^ ^22^, ^23^ ^]^ but had little impact on the metabolic pathway genes (Figure S6C, Supporting Information). High levels of HSV were detected in the brains of both cGAS‐KO mice and OA‐treated WT mice on day 3 postinfection (Figure 6C). Furthermore, OA treatment and cGAS KO separately led to higher mortality (*p* < 0.0001), but not significantly additively (*p* = 0.2001) (Figure 6D), suggesting that FA might regulate anti‐viral surveillance via cGAS.

We then assessed the impact of diet‐elevated FA on antiviral surveillance. Excessive adipose accumulation reduces the potential to buffer diet‐promoted FFA elevation,^[^ ^25^ ^]^ and obesity (particularly severe and morbid obesity) is highly associated with increased FFAs.^[^ ^7^, ^26^ ^]^ Therefore, to enhance the potential of HFDs to elevate FFAs, mice were fed with an HFD for 12 weeks as described in our previous study (Figure 6E),^[^ ^18^ ^]^ leading to increased weight and white adipose tissue (WAT), as well as elevated FFA levels (Figure 6F; Figure S6D, Supporting Information). In line with the FA injection results, HFD (12 weeks) and cGAS KO decreased HSV‐stimulated IFN production, but not substantially additively (Figure 6G; Figure S6E, Supporting Information). High HSV levels were detected in the brains of both cGAS KO mice and HFD‐treated WT mice (Figure 6H). Additionally, HFD and cGAS KO led to increased mortality, but not significantly additively (*p* = 0.2611) (Figure 6I), suggesting that HFD‐elevated FA reduced cGAS‐mediated antiviral immunity.

These results indicate that FAs reduced cGAS‐mediated antiviral immunity.

### 2.7 FAs Attenuate cGAS‐Mediated Anticancer Immunity

**Figure 7:**
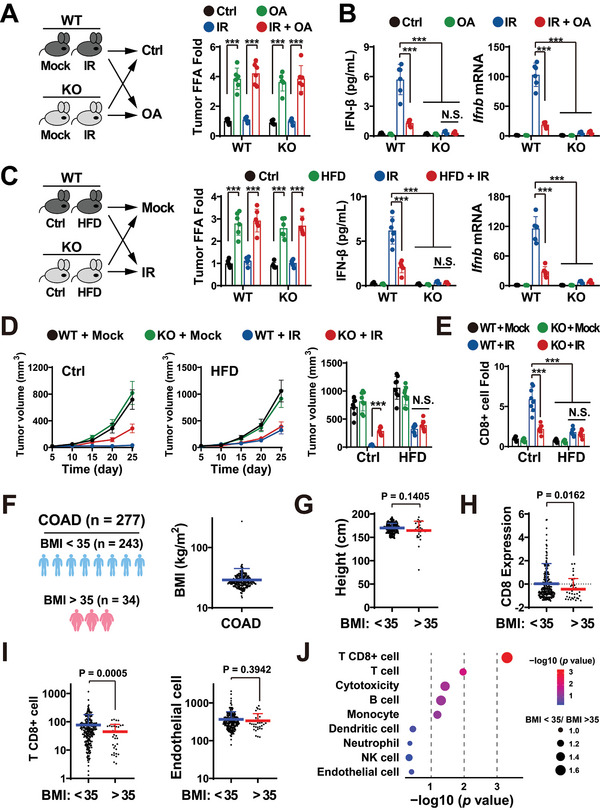
FAs attenuate cGAS‐mediated anticancer immunity. A) Relative FFA levels in the tumors from the mice treated with IR and then OA. Mice were injected subcutaneously with 1 × 10^7^ MC38 cells into the flank. After 10 days, the mice were treated with IP injection of OA at 20 mg per mouse and three focal fractions of 8 Gy IR on three consecutive days. FFA levels in the tumors were analyzed at 12 h post the third treatment of IR and OA. Unless specifically noted, mean ± standard deviation (mean ± SD) was used in this study. Unless specifically noted, *p* values were calculated by unpaired two‐tailed Student's *t*‐test, and *p* values < 0.05 were considered statistically significant (*p < 0.05, **p < 0.01, ***p < 0.001). *n* = 6 mice. B) IFN‐*β* protein and mRNA levels in the tumors described in (A). C) Relative FFA, Ifnb mRNA, and protein levels in the tumors from the mice treated with high fat diet (HFD) and then IR. The mice pretreated with or without HFD for 12 weeks were injected subcutaneously with 1 × 10^7^ MC38 cells. After 10 days, the mice were treated with three focal fractions of 8 Gy of IR on three consecutive days. For HFD groups, the mice were consistently treated with HFD during the experiment. The relative FFA, Ifnb mRNA, and protein levels in the tumors were analyzed at 12 h post the third treatment of IR. *n* = 6 mice. D) MC‐38 tumor volumes in the mice treated with HFD and IR. The mice pretreated with or without HFD for 12 weeks were treated with subcutaneous injection of 1 × 10^7^ MC38 cells. After 5 days, three focal fractions of 8 Gy of IR were used to irradiate the mice on three consecutive days. For HFD groups, the mice were consistently treated with HFD during the experiment. *n* = 8 mice. (C) and (D) were from two independent experiments. E) CD8+ cell levels in the tumors described in (D). F) Body mass indexes (BMIs) in control (BMI < 35, *n* = 243) and severely obese (BMI > 35, *n* = 34) patients with colon adenocarcinoma (COAD). Mean + SD. G) Height in control (BMI < 35, *n* = 243) and severely obese (BMI > 35, *n* = 34) patients with COAD. Mean + SD. H) CD8 expression in the tumors from control (BMI < 35, *n* = 243) and severely obese (BMI > 35, *n* = 34) patients with COAD. The CD8 expression was evaluated by the sum of CD8A and CD8B z‐scores collected from the TCGA. Mean + SD. I) T CD8+ cell (left) and endothelial cell (right) population scores in control (BMI < 35, *n* = 243) and severely obese (BMI > 35, *n* = 34) COAD patients. The scores of cell population abundance were calculated using MCPcounter based on default parameters. Mean + SD. J) Statistical comparisons of the noted cell population abundances between the tumors from control (BMI < 35, *n* = 243) and severely obese (BMI > 35, *n* = 34) patients. *P* values were obtained from unpaired two‐tailed Student's *t*‐test.

Because of the critical role played by the cGAS‐Sting pathway in IR‐promoted anticancer immune surveillance,^[^ ^27^ ^]^ we evaluated the impact of FA on cGAS‐mediated anticancer immunity using immunocompetent syngeneic C57BL/6 mouse models. In this study, cGAS was knocked out in an immunocompetent C57BL/6 mouse line. C57BL/6 mouse cancer cells were used to construct immunocompetent syngeneic models. As previously described,^[^ ^27^, ^28^ ^]^ C57BL/6 mouse MC38 colon cancer cells were subcutaneously injected into immunocompetent cGAS WT and KO C57BL/6 mice. The resultant tumors were treated with three focal fractions of irradiation (IR) on three consecutive days, followed by IP injection of OA. OA treatment elevated FFA levels in the sera (Figure S7A, Supporting Information) and tumors (**Figure** **7**A; Figure S7B, Supporting Information). Notably, OA injection and cGAS KO decreased IR‐stimulated IFN production, but not substantially additively (Figure 7B). Further qRT‐PCR analyses showed that OA diminished the IR‐elevated expression of the downstream genes of the cGAS pathway^[^ ^22^, ^23^ ^]^ but had little impact on the metabolism pathway genes (Figure S7C, Supporting Information). These results suggest that FFA reduces IR‐promoted anticancer immunity via cGAS.

Subsequently, we assessed the influence of HFD on tumor growth in the context of IR. MC38 colon cancer cells were injected subcutaneously into the mice pretreated with or without an HFD for 12 weeks. The resultant tumors were treated with three focal fractions of IR on day 5, 6, and 7. For the HFD groups, mice were treated with HFD during the experiment. As shown in Figure 7C and Figure S7D (Supporting Information), HFD increased tumor FFA levels and decreased IR‐stimulated IFN production. Moreover, HFD and cGAS KO decreased IR‐promoted tumor regression, but not substantially additively (Figure 7D; Figure S7E,F, Supporting Information), raising the role of HFD in cGAS‐dampened tumor growth. HFD and cGAS KO decreased T cell infiltration (Figure 7E; Figure S7G, Supporting Information). We also evaluated the impact of HFD on B16 melanoma and LL/2 lung tumor growth in the context of IR. As shown in Figure S7H,I of the Supporting Information, HFD and cGAS KO decreased B16 melanoma tumor regression and T cell infiltration, but not significantly additively. Similarly, HFD decreased cGAS‐mediated LL/2 lung tumor regression and T cell infiltration (Figure S7J,K, Supporting Information). These results suggest that HFD‐elevated FFA levels might attenuate cGAS‐mediated anticancer immunity.

Additionally, we evaluated the potential impact of FFA on T cell infiltration in human colon tumors. A substantial challenge for this evaluation was the lack of clinical information regarding FFA levels in patients. Fortunately, severe obesity is known to be highly associated with elevated FFA levels.^[^ ^7^, ^26^ ^]^ This strong association allowed for evaluation of the impact of FFA levels by investigating the correlation between severe obesity and T cell infiltration. We then analyzed the colon adenocarcinoma (COAD) RNA‐seq dataset from The Cancer Genome Atlas (TCGA) with corresponding body mass index (BMI) data (*n* = 277, Table S2, Supporting Information). The mean BMI of these COAD patients was ≈29 (Figure 7F), consistent with the impact of excess body weight on cancer morbidity.^[^ ^9^ ^]^ According to the World Health Organization (WHO), a BMI > 35 kg m^−2^ is classified as severe obesity. As shown in Figure 7G, there was no significant difference between the height for the control (BMI < 35) and severe obesity (BMI > 35) patients. However, CD8 mRNA expression was lower in tumors from severely obese (BMI > 35) patients (Figure 7H), consistent with the earlier‐mentioned results for implanted tumors. The abundance of intratumor cell populations was estimated using MCPcounter.^[^ ^29^ ^]^ In line with CD8 expression, MCPcounter showed that T cell (particularly T CD8+ cell) abundances were considerably lower in tumors from severely obese (BMI > 35) patients (Figure 7I,J; Figure S7L, Supporting Information). Endothelial cell abundance did not differ significantly between the control (BMI < 35) and severe obesity (BMI > 35) groups (Figure 7I,J; Figure S7L, Supporting Information). Owing to the association between FFAs and severe obesity, these results at least partially support our results regarding the role of FAs in cGAS‐mediated anticancer immunity.

## 3 Discussion

PS is crucial for life processes including immunity.^[^ ^1^ ^]^ Despite numerous studies on PS, its dissolution is less understood. The present study illustrates that FAs dissolve cGAS–DNA PS to inhibit the immunosurveillance of DNA, which may be targeted for therapeutic intervention.

Our results build a link between metabolism and immunity via PS. The activation of immune sensors should be effectively and efficiently tuned to mount immune responses and diminish harmful autoimmunity.^[^ ^2^ ^]^ As PS can robustly regulate molecule–molecule interaction and molecule concentrations, immune sensors undergo PS to regulate immunity.^[^ ^1^, ^5^ ^]^ This is considerably supported by the unique structure of cGAS–DNA complex.^[^ ^10^, ^11^ ^]^ Unlike other DNA sensors, one cGAS molecule needs to bind two condensed dsDNA strands to activate surveillance.^[^ ^10^, ^11^ ^]^ Importantly, the condensation of negatively charged DNA molecules is energetically unfavorable.^[^ ^12^ ^]^ PS is potent in concentrating molecule(s),^[^ ^5^, ^10^, ^11^ ^]^ raising its role in cGAS activation.^[^ ^5a^ ^]^ PS is controlled by solvent components, and metabolites are abundant cellular components,^[^ ^6^ ^]^ suggesting that metabolites play a role in PS‐regulated cellular processes such as immune activation. However, little is known about whether, which, and how metabolites regulate immunity via PS. The present study shows that FAs control the PS of immune sensors to regulate surveillance. Notably, HFD and metabolic syndrome worsen numerous diseases by deregulating immunity.^[^ ^7^, ^8^ ^]^ Owing to metabolite abundance and PS potential,^[^ ^1^, ^6^, ^30^ ^]^ metabolites might regulate immune processes via PS. Further study is required to identify the metabolites that regulate immunity via PS.

This study reveals the role of metabolites in PS dissolution. Owing to the notable impact of PS, intense studies have focused on PS. However, little is known about the physical dissolution of PS, which is the reverse of the PS. The present study shows that FAs dissolve cGAS–DNA PS. As a physicochemical phenomenon, PS is controlled by the solvent components.^[^ ^1b^ ^]^ FAs are abundant in cellular components,^[^ ^6^ ^]^ and contain a charged carboxylate hydrophilic end and a hydrocarbon hydrophobic end, which enables them to disperse one liquid into another immiscible liquid to dissolve the condensates formed via PS.^[^ ^17^ ^]^ Consistently, soaps (FA salts) have been widely used for centuries.^[^ ^17^ ^]^ Given the diversity of PS that soaps (the salts of FA) can dissolve, FAs may contribute to other physiological and pathological processes by dissolving the corresponding PS, and identification of the cellular PS dissolved by FAs would be of interest.

Both cGAS and FAs are pivotal in multiple diseases including aging, cancer, infection, and autoimmune diseases.^[^ ^7^, ^13^ ^]^ Here, we show that FAs dissolve cGAS–DNA PS to attenuate antiviral and antitumor immunity. Because HFDs and metabolic syndromes are well‐documented to elevate FFA levels, our findings provide a potential explanation for the potential of HFDs and metabolic syndromes to worsen diseases.^[^ ^7^, ^8^ ^]^ Given the roles of cGAS, these results suggest possible metabolic information to improve therapeutic strategies and patient prognostication. For instance, targeting FAs might enhance cGAS‐promoted antiviral and antitumor immune surveillance, particularly in patients with metabolic syndromes. Conversely, FA supplementation may benefit the treatment of cGAS‐mediated autoimmune or autoinflammatory diseases such as Aicardi–Goutières syndrome.^[^ ^13^ ^]^

However, this study had several limitations. First, although FAs decrease the cGAS activation by DNA, we did not exclude the possibility that FAs regulate other sensors. Owing to the central role of PS in DNA‐sensing,^[^ ^1^, ^5^ ^]^ FAs might reduce the activation of other DNA sensors. Second, our study focused on cGAS‐mediated antiviral and anticancer surveillance. Because cGAS is critical to multiple autoimmune diseases, FAs might also be involved in autoimmunity by dissolving cGAS–DNA PS. Third, to mimic the in vivo context, OA was used to evaluate the effect of FAs. However, certain FA(s) might also regulate immunity in a cGAS‐independent manner, which might lead to different or converse results. Despite these limitations, our study still reveals the role of metabolites in immunity by dissolving PS.

## 4 Experimental Section

### Cell Culture

MC38, B16, LL/2, U2OS, and Vero cells were maintained in Dulbecco's modified Eagle's medium containing 10% fetal bovine serum (FBS), 100 U mL^−1^ penicillin, and streptomycin at 37 °C under a humidified atmosphere of 5% CO_2_. THP‐1 cells were maintained in RPMI‐1640 medium (Thermo Fisher Scientific Inc., Waltham, MA, USA) containing 10% FBS. THP‐1 cell differentiation was induced by treatment with 0.1 mm phorbol 12‐myristate 13‐acetate (PMA) for 24 h. MEF cells were prepared and maintained as previously described.^[^ ^14^, ^23^ ^]^ HSV was propagated and titrated in Vero cells. DNA transfection was performed using Lipofectamine 2000 (Thermo Fisher Scientific Inc., Waltham, MA, USA) according to the manufacturer's instructions.

### Reagents

Anti‐ACTIN (Cat. number: 4967), anti‐IRF3 (Cat. number: 4947), anti‐phospho‐IRF3 (Ser396) (Cat. number: 83611), antihuman cGAS (Cat. number: 15102), and antimouse cGAS (Cat. number: 31659) antibodies were purchased from Cell Signaling (Beverly, MA, USA). Anti‐dsDNA (Cat. number: LS‐B6572) and anti‐SC35 (Cat. number: S4045) antibodies were obtained from LifeSpan BioSciences (Seattle, WA, USA) and Sigma‐Aldrich (Saint Louis, MO, USA), respectively. Anti‐PML (Cat. number: sc‐966) antibody was purchased from Santa Cruz Biotechnology (Dallas, TX, USA). Anti‐LSM14A (Cat. number: 18336‐1‐AP) and anti‐G3BP1 (Cat. number: 66486‐1‐lg) antibodies were obtained from Proteintech (Wuhan, Hubei, China).

The full‐length human cGAS (NM_138441.3) sequence was cloned into the Nco I and Sal I sites of the pET‐28a vector (Novogen Limited, Hornsby Westfield, NSW, Australia) to construct the pET‐28a‐cGAS (WT) plasmid for the expression of cGAS protein. Human cGAS (NM_138441.3) amino acid 1–160 and 161–522 sequences were cloned into the pET28a vector to construct the pET‐28a‐cGAS‐N and pET‐28a‐cGAS‐C truncated mutant plasmids, respectively. All lysine (K) and arginine (R) residues of human cGAS‐N (amino acid 1–160) were replaced with neutral alanine (A) to generate the pET‐28a KR–A cGAS‐N mutant plasmid using synthetic oligo DNA. Full‐length human cGAS was cloned into the Xba I and Not I sites of the pCDH‐CMV‐MCS‐EF1‐Puro vector (System Biosciences, Mountain View, CA, USA) to construct the pCDH Flag cGAS‐FL plasmid. Human cGAS (NM_138441.3) amino acid 1–160 and 161–522 sequences were cloned into the Xba I and Not I sites of the pCDH‐CMV‐MCS‐EF1‐Puro vector to construct the pCDH Flag cGAS‐N and pCDH Flag cGAS‐C truncated mutant plasmids, respectively. Full‐length mouse cGAS (NM_173386.5) was cloned into the Nhe I and Xho I sites of the pET28a vector to construct the pET‐28a‐mcGAS plasmid. IFN‐*α* (Cat. number: 447904) and IFN‐*β* (Cat. number: 439407) enzyme linked immunosorbent assay (ELISA) kits were purchased from Biolegend (San Diego, CA, USA). Free Fatty Acid Quantification Kit (Cat. number: MAK044) and fatty‐acid‐free bovine serum albumin (BSA) (Cat. number: A7030) were obtained from Sigma‐Aldrich (St. Louis, MS, USA). cGAS inhibitor, G150 (Cat. number: HY‐128583), was purchased from MedChemExpress (Shanghai, China). ATP (Cat. number: R0441), GTP (Cat. number: R046), Alexa Fluor 488 (AF488) and Cy5 Protein Labeling Kits were obtained from ThermoFisher Scientific (Waltham, MA, USA). 2′3′‐cGAMP (Cat. number: tlrl‐nacga23) and Pronase (Cat. number: 10165921001) were purchased from InvivoGen (San Diego, CA, USA) and Roche (Risch‐Rotkreuz, Switzerland), respectively.

### Expression and Purification of cGAS Proteins

Summarily, *Escherichia coli* strain, BL21 (DE3), was separately transformed with the plasmids encoding the noted cGAS proteins. cGAS proteins were expressed and purified as described in the previous study.^[^ ^19^ ^]^ Concentration of the resultant proteins and buffer exchange were performed using an Amicon Ultrafree centrifugal filter (Millipore) with a cutoff of 10 kDa. The proteins were labeled with Alexa Fluor 488 (AF488) or Cy5 using Protein Labeling Kits (ThermoFisher Scientific, Waltham, MA, USA). The estimated degree of labeling was 2 mol of Alexa Fluor 488 or Cy5 per mol of protein.

### In Vitro cGAS–DNA Phase Separation Assay

cGAS–DNA phase separation assays were performed as described in the previous study.^[^ ^19^ ^]^ 20 µm of noted cGAS protein was mixed with 20 µm of DNA in a buffer containing 20 mm Tris‐HCl (pH 7.5) and 150 mm NaCl at 37 °C for 15 min. The resultant mixtures were incubated with 500 µm of the metabolite at 37 °C for 15 min. Turbidity was assessed at 340 nm using NanoDrop 2000C (Thermo Scientific, Waltham, MA, USA). For fluorescence analyses, AF488‐labeled cGAS protein was mixed with Cy3‐labeled 45‐bp ISD (DNA) in a buffer containing 20 mm Tris‐HCl (pH 7.5) and 150 mm NaCl. After incubation for the indicated times, fluorescent images of the mixtures were captured by fluorescence microscopy (Olympus IX81, Japan).

### Metabolite Affinity Responsive Target Fluorescence Quenching Assay

MARTFQ experiments were performed as described in the previous studies.^[^ ^18^, ^19^ ^]^ Briefly, 50 nm cGAS protein was mixed with serial dilutions of metabolites as noted. After incubation for 5 min, the samples were subjected to fluorescence analyses based on 348 nm intrinsic protein fluorescence excited by 280 nm light. The binding constants were estimated according to the modified Stern–Volmer equation: RF0/ΔRF = (1/*fK*) × (1/[*Q*]) + (1/*f*), where ∆RF is equal to RF0 (the protein fluorescence intensity in the absence of metabolite) – RF (the intensity in the presence of metabolite), *f* is the fractional maximum fluorescence intensity of the protein, *K* is the quenching constant, considered as the binding constant,^[^ ^31^ ^]^ and [*Q*] is the concentration of the metabolite.

### Drug Affinity Responsive Target Stability Assay

DARTS assays were performed as described in the previous studies.^[^ ^18^, ^19^ ^]^ Briefly, the lysates from the MEF cells pretransfected with or without the noted plasmid(s) were incubated with FA for 1 h. Pronase (a protease) was then added to the mixture at 1:2000, followed by incubation at room temperature for 30 min. Digestion was stopped by adding sodium dodecyl sulfate (SDS) loading buffer and heating at 95 °C for 10 min. The resultant mixtures were subjected to immunoblotting with antibodies.

### Cellular Thermal Shift Assay

CETSAs were performed as described in the previous studies.^[^ ^18^, ^19^ ^]^ Summarily, MEF cells were treated with 500 µm FA(s) for 2 h. The resultant cells were heated at the indicated temperatures for 3 min and the cell lysates were subjected to immunoblotting with an anti‐cGAS antibody. Immunoblotting band intensities were quantified using Gel‐Pro analyzer software and plotted against temperatures.

### Circular Dichroism Assay

CD assays were performed as described in the previous study.^[^ ^19^ ^]^ Summarily, CD spectra (180–260 nm) of 5 µm cGAS in 500 µm FA were measured using a Chirascan spectrometer at room temperature.

### Microscale Thermophoresis Assay

MST assays were performed as described in the previous study.^[^ ^19^ ^]^ Summarily, 50 nm Cy5‐labeled DNA or 100 nm Cy5‐labeled cGAS protein was mixed with the noted concentration of cGAS protein and/or FA(s) in a buffer containing 20 mm Tris‐HCl (pH 7.5) and 150 mm NaHCO_3_ (pH 7.6). The thermophoretic movements of DNA or protein were analyzed using a Monollith NT.115 instrument (Nanotemper Technologies).

### Electrophoretic Mobility Shift Assay

EMSAs were performed as described in the previous study.^[^ ^19^ ^]^ Summarily, 50 nm Cy3‐labeled DNA was mixed with the noted concentration of cGAS and/or FA in a buffer containing 20 mm Tris‐HCl (pH 7.5) and 150 mm NaCl. After incubation at 37 °C for 10 min, the mixtures were analyzed on a 1.5% agarose gel. Images were acquired using ChemiDoc XRS+ (Bio‐Rad) and analyzed by ImageJ.

### Immunoblotting Analysis

Immunoblotting was performed as previously described.^[^ ^32^ ^]^ Summarily, cells were harvested in RIPA lysis buffer containing 0.1% SDS, 0.1 mg mL^−1^ phenylmethanesulfonyl fluoride, 1 mm sodium orthovanadate, 1 × phosphate‐buffered saline (PBS), 1% NP40, 0.5% sodium deoxycholate, protease inhibitor cocktail (Roche, Indianapolis, IN, USA), and phosphatase inhibitor cocktail (Roche, Indianapolis, IN, USA). The resultant cell lysates were resolved by SDS–polyacrylamide gel electrophoresis and blotted with the indicated antibodies.

### Immunofluorescent Analysis

IF analyses were carried out as previously described.^[^ ^18^ ^]^ Summarily, cells were plated on the coverslips precoated with type I collagen. After the indicated treatments, the cells were fixed with 4% formaldehyde in PBS for 10 min at room temperature and permeabilized with 0.2% Triton‐X 100 in PBS for 10 min. The resultant cells were incubated with rabbit (or mouse) primary antibody for 1.5 h, followed by incubation with the fluorescence‐labeled goat anti‐rabbit (or anti‐mouse) immunoglobulin G (IgG) (Thermo Fisher Scientific Inc., Waltham, MA, USA) for 1 h. Subsequently, the cells were incubated with mouse (or rabbit) primary antibody for 1.5 h. After incubation with fluorescence‐labeled goat anti‐mouse (or anti‐rabbit) IgG for 1 h, the coverslips were mounted with a drop of mounting medium supplemented with 4′,6‐diamidino‐2‐phenylindole (DAPI) (Vector Laboratories, Burlingame, CA, USA), and then sealed with clear nail polish.

### Immunoprecipitation

Immunoprecipitation (IP) assays were performed as previously described.^[^ ^33^ ^]^ Summarily, MEF and U2OS cytosolic lysates were mixed with 10 nm biotin‐labeled ISD and/or 1 mm OA for 30 min. The mixtures were then incubated with streptavidin‐coupled Dynabead at 4 °C overnight. The precipitates were denatured in SDS protein sample buffer at 95 °C and resolved by SDS–polyacrylamide gel electrophoresis.

### Quantification of cGAMP by Liquid Chromatography‐Mass Spectrometry/Mass Spectrometry

Quantification of cGAMP by liquid chromatography‐mass spectrometry/mass spectrometry (LC‐MS/MS) was performed as described in the previous study.^[^ ^19^ ^]^ The samples were prepared as previously described.^[^ ^34^ ^]^ The resultant samples were separated using the ACQUITY UPLC system (Waters), followed by MS detection using a Qtrap5500 triple quadrupole mass spectrometer (ABSciex) in the positive electrospray ionization mode. The multiple‐reaction‐monitoring mode was used to monitor the cGAMP parent ion to product ions: m/z 675 to 136 (collision energy (CE) 45 V; declustering potential (DP) 70 V), m/z 675 to 506 (CE 98 V; DP 31 V), and m/z 675 to 524 (CE 90 V; DP 33 V). And m/z 675 to 136 was used for the quantification of cGAMP levels according to the standard curve. The mobile phases (delivered at 0.45 mL min^−1^) consisted of H_2_O (containing 20 mm NH_4_Ac and 0.05% HAc) for A and methanol (containing 20 mm NH_4_Ac and 0.05% HAc) for B. Isocratic elution (50% A and 50% B) was performed for 5 min. The Analyst software for Windows (Version, 1.6.1) was used for peak area quantification and data processing.

### cGAS Activity Assay

cGAS activity assays were performed as described in the previous study.^[^ ^19^ ^]^ Briefly, in the presence of the noted compound, cGAS proteins at the noted concentrations were mixed with DNA in a buffer containing 20 mm Tris‐HCl (pH 7.5), 100 mm NaCl, 1.5 mm MgCl_2_, 2 mm ATP, and 0.5 mm GTP. After incubation at 37 °C for 30 min, the mixture was diluted and centrifuged at 16 000 × *g* for 10 min. The resultant supernatants were analyzed using liquid chromatography with tandem LC–MS/MS as described above.

### Measurement of Kinetic Parameters of cGAS

Kinetic parameters were determined by varying the ATP substrate concentration and maintaining the constant concentrations of GTP, DNA, cGAS, and FA. Summarily, a serial dilution of ATP was mixed with 20 mm Tris‐HCl (pH 7.5), 100 mm NaCl, 1.5 mm MgCl_2_, 0.5 mm GTP, 200 nm DNA, 200 nm cGAS, and 50 µm noted FA. Distilled water was used as control. After incubation at 37 °C for 5 min, the produced cGAMP levels were assessed using MS analysis. The initial reaction velocity (µm min^−1^) at each substrate concentration was calculated from the plot of cGAMP production versus reaction time. All reactions were performed in triplicate and analyzed using GraphPad software with the Michaelis–Menten equation.

### Fluorescence Polarization Assay

FP assays were performed as previously described.^[^ ^35^ ^]^ Summarily, 100 µm BODIPY‐labeled OA and a serial dilution of cGAS were mixed at room temperature for 5 min. The 510 nm fluorescence of the resultant mixtures was measured using a Biotek Synergy Neo plate reader. The assays were performed in triplicate and analyzed using the GraphPad software with the one‐site binding model.

### Isothermal Titration Calorimetry

ITC was performed as previously described.^[^ ^36^ ^]^ Briefly, the titration of OA to cGAS protein was performed at 25 °C (298 K) using a MicroCal Auto‐ITC 200 system (GE Healthcare). cGAS and OA stock solutions were diluted to 10 µm and 3 mm, respectively, using a buffer containing 1 mm Tris pH 7.0. 190 µL cGAS protein was injected into the sample cell. ITC titration experiments were carried out by injecting 19 × 2 µL of OA into the cGAS protein solution, spaced at 200 s intervals. The stirring rate and reference cell power were set to 150 rpm and 5 µcal s^−1^, respectively. Kd, molar ratio, Gibbs free energy, enthalpy, and entropy of cGAS–OA binding were calculated using MicroCal analysis software.

### Prediction of the Isoelectric Points (pIs) of the cGAS Proteins

The pI values of cGAS proteins were predicted by Compute pI/Mw at ExPASy (https://web.expasy.org/compute_pi/).^[^ ^37^ ^]^

### Quantification of Free Fatty Acids

Free fatty acid levels were quantified using a free fatty acid quantization kit from Sigma‐Aldrich according to the manufacturer's instructions.

### Real‐Time Quantitative Reverse Transcription PCR

Real‐time qRT‐PCR was performed as described in the previous study.^[^ ^18^, ^19^ ^]^ Summarily, RNA was extracted using RNeasy Mini Kit (QIAGEN, Hilden, Germany). Reverse transcription was performed using PrimeScript RT Reagent Kit (TaKaRa, DaLian, Liaoning, China). qRT‐PCR was performed using SYBR PrimeScript PCR Kit II (TaKaRa, DaLian, Liaoning, China). Primers used for qRT‐PCR are listed in Table S3 of the Supporting Information.

### Enzyme Linked Immunosorbent Assay

Summarily, ELISAs were performed using ELISA kits from BioLegend (San Diego, CA, USA) as described in the previous study.^[^ ^19^ ^]^ The cell culture supernatants or sera were mixed with 50 µL of Assay Buffer A at room temperature for 2 h. Then, 100 µL of the appropriate antibody solution was added, followed by incubation at room temperature for 2 h. After washing, 100 µL avidin‐HRP D solution was added and incubated at room temperature for 30 min. After washing, 100 µL Substrate Solution F was added to each well, followed by incubation for 30 min in the dark. The reactions were stopped by adding 100 µL Stop Solution. The absorbance (450 nm) of each sample was measured, and IFN‐*α* and IFN‐*β* protein levels were quantified according to standard curves.

### Mice

Animal handling procedures were approved by the Animal Care and Use Committee of DaLian Medical University (Approval number: AEE22078). All mice were housed in a specific pathogen free facility at 22 ± 2 °C under a cycle of 12 h light (7:00 am light on) and 12 h dark (7:00 pm light off). To create a cGAS knockout (KO) mouse model (C57BL/6) using CRISPR/Cas‐mediated genome‐editing technology, a construct resulting was designed in the deletion of 2–4 exons of the cGAS coding sequence, which resulted in a cGAS frameshift. Cas9 mRNA and sgRNA generated by in vitro transcription were injected into fertilized eggs for KO mouse productions. The founders were genotyped by PCR (F1: 5′‐TATGTACAGGAACCCGTGCAG‐3′; R1: 5′‐CTTAACCACTGAGCCATCTCTAG‐3′), followed by DNA sequencing. The positive founders were bred to the next generation (F1) and subsequently genotyped by PCR (F2: 5′‐TTCACTAAATAGACCAAGCTGCTG‐3′; R2: 5′‐ATGACTCAGCGGATTTCCTCG‐3′), DNA sequencing and immunoblotting.

### High Fat Diet Treatment

HFD treatment was performed as described in the previous study.^[^ ^18^ ^]^ Briefly, mice were fed with standard rodent chow (Ctrl) or a HFD (60 kcal% fat diet, D12492, Research Diets) ad libitum for the noted time. Body weight and WAT analyses were performed as described in the previous study.^[^ ^18^ ^]^ Mice treated with a HFD for 12 weeks were used for the further HSV infection and tumor challenge. For FA treatment, mice were intraperitoneally (IP) injected with 20 mg per mouse OA (OA: fatty‐acid‐free BSA = 5:1), as previously described.^[^ ^38^ ^]^

### HSV Infection

HSV infection was performed as previously described.^[^ ^14c^ ^]^ To measure IFN production, 5–8‐week‐old mice were infected intravenously (IV) with HSV at 1 × 10^7^ plaque‐forming units (pfu) per mouse, and sera were collected to measure IFN levels. For the brain viral titer and survival experiments, mice were infected IV with 1 × 10^6^ pfu HSV per mouse. The brains of the infected mice were excised on day 3 to assess viral titers via plaque assay. Mouse survival was monitored for 7 days.

### Tumor Challenge and Treatment

Tumor challenge and treatment were performed as previously described.^[^ ^27^, ^39^ ^]^ Summarily, 5–8‐week‐old mice were injected subcutaneously (s.c.) with 1 × 10^7^ noted cancer cells in the right flank. On day 5 and 10, the mice were randomly grouped as indicated. IR was performed using an X‐RAD320 Small Animal Irradiator (Precision X‐Ray, North Branford, CT, USA). Three focal fractions of 8 Gy IR were used to irradiate the mice on three consecutive days. Tumor size was measured using calipers, and tumor volumes were calculated using the formula: 0.52 × *L* × *W* ^2^, where *L* was the longest diameter and *W* was the shortest diameter.

### Bioinformatics Analyses

Bioinformatics analyses were performed as described in the previous study.^[^ ^40^ ^]^ Briefly, TCGA data for colorectal adenocarcinoma regarding gene expression and clinical information were collected using The cBio Cancer Genomics Portal (http://cbioportal.org).^[^ ^41^ ^]^ Only samples (*n* = 277) with both gene expression and clinical information were used for further analyses (Table S2, Supporting Information). BMI was calculated as patient weight/height^2^. As described by the World Health Organization (WHO), a BMI of 35 was used as the cut‐off point for the severely obese patients. CD8 expression was evaluated using the sum of CD8A and CD8B z‐scores, which were collected from TCGA.^[^ ^41a^ ^]^ The z‐scores for gene expression were calculated for each sample by comparing the RNA expression of a gene to the distribution in a reference population, which represents the typical expression of the gene. The intratumor cell population abundance was estimated using the MCPcounter R‐package (http://github.com/ebecht/MCPcounter)^[^ ^29^ ^]^ using default parameters.

### Statistical Analyses

All statistical tests, normalizations, comparisons, replications, and sample sizes are presented in the figure legends. All data are presented as mean ± standard deviation (mean ± SD). Error bars indicate standard deviation (SD). For Kaplan‐Meier analyses, the log‐rank test was used to calculate *p* values. All other statistical analyses were performed using unpaired two‐tailed Student's *t*‐test. In all cases, ***p < 0.001, **p < 0.01, *p < 0.05, and N.S. = not statistically significant (*p* > 0.05). The experiments were replicated at least three times, and all attempts at replication were considered successful. All statistical analyses were performed using Prism 9 software (GraphPad Software).

## Conflict of Interest

The authors declare no conflict of interest.

## Supporting information

Supporting Information

## Data Availability

The data that support the findings of this study are available in the supplementary material of this article.
