# Supplementary material for: Oleic Acid Dissolves cGAS–DNA Phase Separation to Inhibit Immune Surveillance
Source: Adv Sci (Weinh). 2023 Mar 22;10(14):2206820. doi: 10.1002/advs.202206820 (PMC10190586; doi:10.1002/advs.202206820)
Supplement: Supplementary file 1 — Supporting Information [file ADVS-10-2206820-s001.pdf]

ADVANCED SCIENCE
Open Access

Supporting Information

for *Adv. Sci.*, DOI 10.1002/advs.202206820

Oleic Acid Dissolves cGAS–DNA Phase Separation to Inhibit Immune Surveillance

Lina Wang, Qiaoling Liu, Na Wang, Siru Li, Wei Bian, Zhen Sun, Lulu Wang, Li Wang, Caigang Liu, Chengli Song, Quentin Liu* and Qingkai Yang**

Supporting Information:

Figure S1. Metabolite screening revealed that FAs dissolve the cGAS–DNA phase separation.

A) Coomassie staining of the *in vitro* purified full-length human cGAS protein. M: marker; kD: kilodalton.

B) Representative micrographs of fluorescence recovery after photobleaching (FRAP) experiments. 20 µM of AF488-cGAS was mixed with 20 µM of Cy3-DNA at 37°C for 15 min, at which time a laser was used to photobleach the condensates formed via PS. Time 0 indicates the end of the photobleaching pulse and the start of recovery at 37°C. Green: AF488-labeled cGAS; Red: Cy3-labeled ISD DNA. Scale bar = 5 µm.

C) FRAP of the cGAS–DNA condensates treated as described in (B) was measured by Cy3-DNA fluorescence intensities. n = 3.

D) Representative fluorescent images of the mixtures of cGAS protein, DNA and the noted metabolite. 20 µM of cGAS protein, 20 µM of DNA and 500 µM noted metabolite were simultaneously mixed and then incubated for 15 min. ALA: α-linolenic acid; LA: linoleic acid; OA: oleic acid; AcOH: acetic acid; ACAC: acetoacetic acid; Hex: 1,6-hexanediol; 1000×Hex: 0.5 M of 1,6-hexanediol. Green: AF488-labeled cGAS; Red: Cy3-labeled ISD; BF: bright field. Scale bar = 10 µm. n = 3.

E) Representative fluorescent images of the condensate dissolution by OA for different times. 20 µM AF488-cGAS and 20 µM Cy3-ISD were mixed for 15 min. Then, the mixtures were incubated with 500 µM of OA for the noted times. Green: AF488-labeled cGAS; Red: Cy3-labeled ISD; BF: bright field. Scale bar = 5 µm. n = 3.

F) Turbidity analyses of the condensate dissolution by FAs for different times. 20 µM cGAS protein was mixed with 20 µM ISD for 15 min. After the incubation with 500 µM FA for the noted times, the turbidities of the mixtures were measured at 340 nm. n = 3.

G) Coomassie staining of the *in vitro* purified full-length mouse cGAS (mcGAS) protein. M: marker; kD: kilodalton.

`

H) Turbidity analyses of the dissolution of mcGAS–DNA condensates by the noted FAs. 20 µM mcGAS protein was mixed with 20 µM ISD for 15 min. Then, the mixtures were incubated with the noted concentration of FA for 15 min. n = 3.

I) Representative fluorescent images of the dissolution of mcGAS–DNA condensates by the noted metabolites. 20 µM AF488-labeled mcGAS protein was mixed with 20 µM Cy3-labeled ISD for 15 min. Then, the mixtures were incubated with 500 µM metabolite for 15 min. Green: AF488-mcGAS; Red: Cy3-ISD; BF: bright field. Scale bar = 10 µm. n = 3.

J) Representative fluorescent images of the mixtures of mcGAS protein, DNA and the noted metabolite. 20 µM mcGAS protein, 20 µM ISD and 500 µM noted metabolite were simultaneously mixed and then incubated for 15 min. Green: AF488-mcGAS; Red: Cy3-ISD; BF: bright field. Scale bar = 10 µm. n = 3.

Figure S1

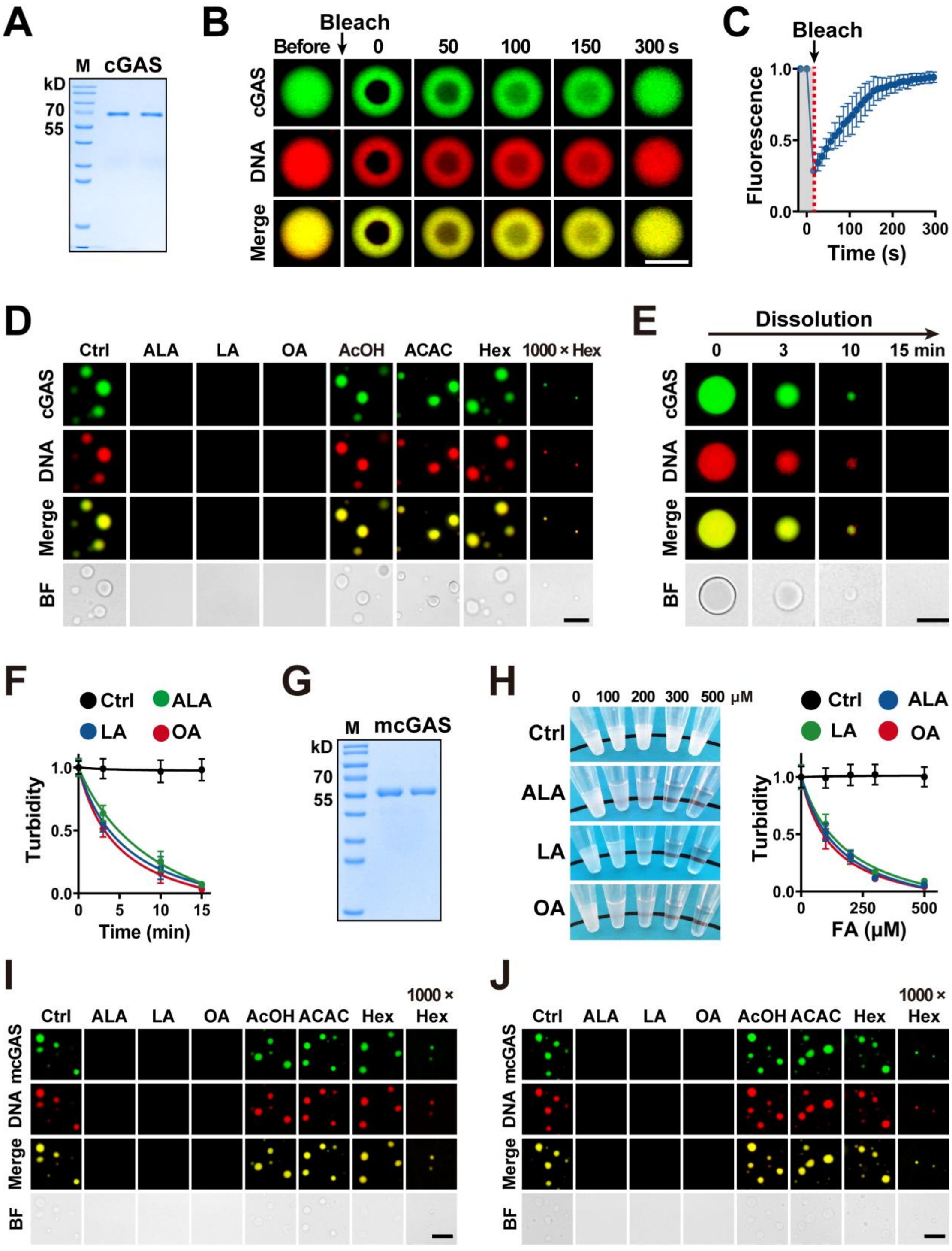
A
M
cGAS
kD
70
55
B
Bleach
Before
0
50
100
150
300 s
cGAS
DNA
Merge
C
Bleach
Fluorescence
Time (s)
D
Ctrl
ALA
LA
OA
AcOH
ACAC
Hex
1000 × Hex
BF
E
Dissolution
0
3
10
15 min
F
Turbidity
Time (min)
G
mcGAS
H
0
100
200
300
500 μM
FA (μM)
I
J
mcGAS

`

Figure S2. FAs inhibit the cGAS binding and activation by DNA *in vitro.*

A) MARTFQ analyses of the quenching of cGAS fluorescence by the serial dilutions of noted metabolites. GTP: guanosine triphosphate; ALA: α-linolenic acid; LA: linoleic acid; OA: oleic acid. *n* = 3.

B) Circular dichroism (CD) analyses of 5 μM of cGAS in 500 μM of the noted FA. *n* = 3.

C and D) Kinetic curves (C) and parameters (D) of cGAS activity for ATP in the context of 45-bp ISD and noted FAs. Kinetic parameters were determined by varying the ATP substrate concentration and maintaining the constant concentrations of DNA (200 nM), cGAS (200 nM) and FA (50 μM). Distilled water was used as control. cGAMP levels were assessed by MS. All reactions were performed at 37°C in triplicate and analyzed by GraphPad software using the Michaelis-Menten equation. *n* = 3.

E and F) Kinetic curves (E) and parameters (F) of cGAS activity for ATP in 50 μM noted FA, 200 nM cGAS and 200 nM herring testis DNA (HT-DNA). Kinetic parameters were determined as described in (C and D). *n* = 3.

G) Electrophoretic mobility shift assays (EMSAs) of mcGAS protein binding to ISD in 200 μM FAs. 200 μM FA and 50 nM ISD were mixed with serial dilutions of mouse cGAS (mcGAS) protein for 30 min. The resultant mixtures were analyzed with 1.5% agarose gel. mcGAS: mouse cGAS. Ctrl: distilled water; ALA: α-linolenic acid; LA: linoleic acid; OA: oleic acid. *n* = 3.

H) EMSA analyses of mcGAS protein binding to ISD in serial dilutions of noted FAs. 50 nM ISD and 2.5 μM mcGAS were incubated with serial dilutions of FAs for 30 min. mcGAS: mouse cGAS. FA: fatty acid; Ctrl: distilled water; ALA: α-linolenic acid; LA: linoleic acid; OA: oleic acid. *n* = 3.

I) Left: MS analyses of the cGAMP produced by the mixing of 50 μM noted FA, 200 nM mcGAS and a serial dilution of ISD for 30 min. Right: cGAMP produced by the mixing of 50 μM noted FA, 200 nM ISD and a serial dilution of mcGAS for 30 min. *n* = 3.

J) Serial dilutions of FA inhibited the cGAMP production in the mixture of 200 nM of mcGAS and 200 nM of ISD for 30 min. FA: fatty acid. *n* = 3.

`

Figure S2

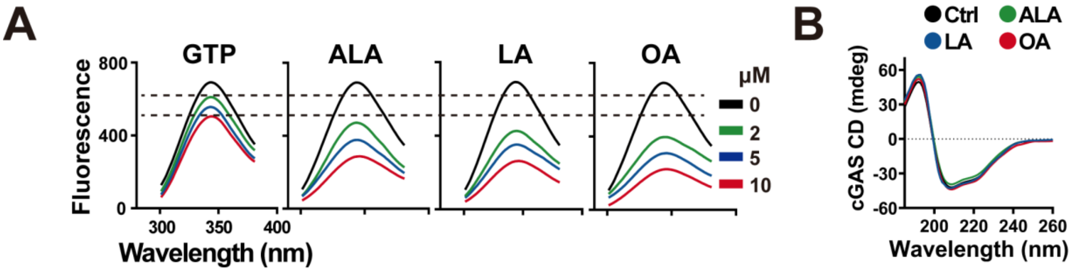

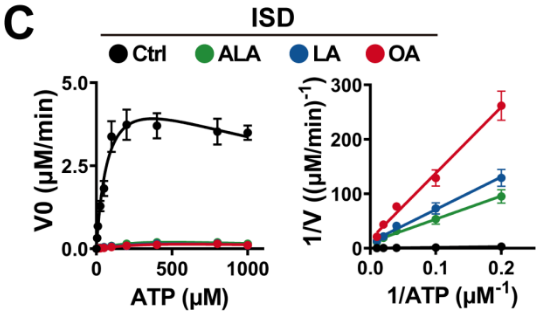

D

	K_{cat} (min^{-1})	K_m (μM)	K_{cat}/K_m (min^{-1} μM^{-1})
Ctrl	28.1 ± 3.3	80.6 ± 22.3	0.35
ALA	4.3 ± 0.6	873.5 ± 265.5	0.0049
LA	3.2 ± 0.5	989.5 ± 295.3	0.0032
OA	2.6 ± 0.4	1076.4 ± 311.2	0.0024

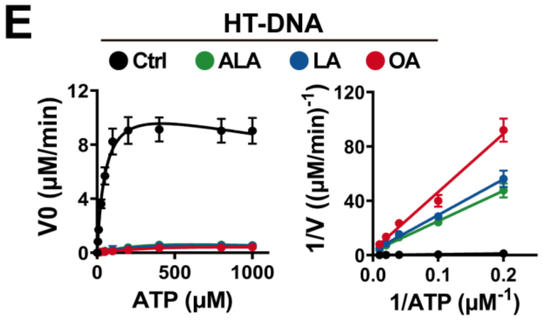

F

	K_{cat} (min^{-1})	K_m (μM)	K_{cat}/K_m (min^{-1} μM^{-1})
Ctrl	63.5 ± 7.8	54.2 ± 13.7	1.171
ALA	5.6 ± 1.1	773.5 ± 231.8	0.0072
LA	5.1 ± 0.9	809.5 ± 255.3	0.0063
OA	3.4 ± 0.7	876.4 ± 261.8	0.0039

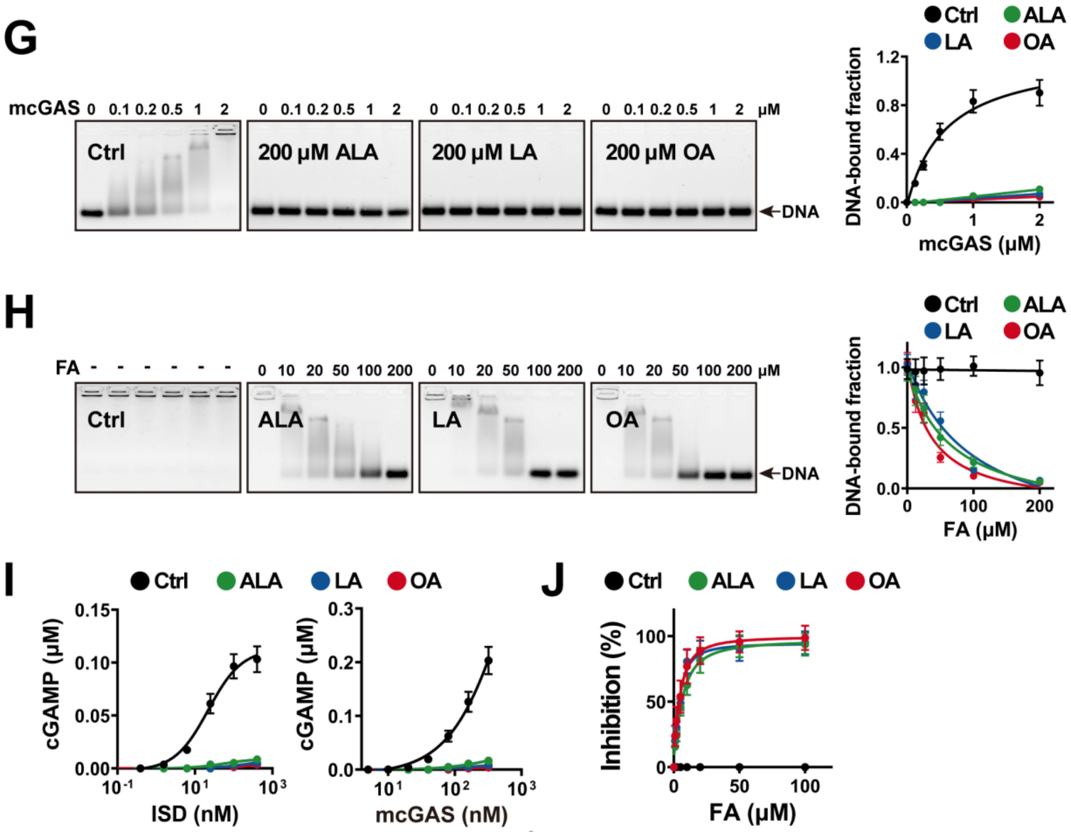

Figure S3. cGAS–OA binding might be regulated by pH.

A) Fluorescence polarization (FP) analyses of OA binding to cGAS protein. 100 µM BODIPY™-labeled OA were mixed with a serial dilution of cGAS for 5 min. Then, the 510 nm fluorescence of the mixtures was measured. mP: millipolarization. *n* = 3.

B) Isothermal titration calorimetry (ITC) titration of OA to cGAS protein. ITC assays were performed as described in the Experimental Section. Left: Enthalpy (ΔH) values are plotted against the molar ratio of OA/cGAS. Right: Obtained ITC parameters. *n* = 3.

C) Coomassie staining of purified N- and C- terminal truncated human cGAS proteins. cGAS-N: N-terminal cGAS protein; cGAS-C: C-terminal cGAS protein.

D) Representative images of the dissolution of cGAS–DNA condensates by OA. 20 µM ISD was mixed with 20 µM cGAS-FL, cGAS-N or cGAS-C protein for 15 min. Then, the mixtures were incubated with 500 µM OA for 15 min. Green: AF488-cGAS proteins; Red: Cy3-ISD; BF: bright field. Scale bar = 10 µm. *n* = 3.

E and F) EMSA analyses of cGAS-N (C) and cGAS-C (D) proteins binding to DNA in OA. 100 µM OA and 50 nM ISD were incubated with a serial dilution of cGAS-N or cGAS-C protein for 30 min. Resultant mixtures were analyzed with agarose gel. Ctrl: distilled water; OA: oleic acid. *n* = 3.

G) Surface structure of cGAS (Protein Data Bank: 4LEZ) to show the DNA-binding residues. Fifteen basic DNA-binding residues (arginine and lysine) are shown in red, while the only one serine DNA-binding residue (S165) is shown in blue.

H) The predicted isoelectric point (pI) of cGAS proteins. The pI values of cGAS proteins were predicted by Compute pI/Mw at ExPASy using the default parameters.

I) EMSA analyses of cGAS proteins binding to DNA at the distinct pH. At the noted pH, 50 nM of ISD was incubated with 2.5 µM cGAS-FL, 20 µM GAS-N or 40 µM cGAS-C protein for 30 min. Resultant mixtures were analyzed by agarose gel. *n* = 3.

J) DARTS analyses of cGAS binding to OA at the noted pH. MEF cells were transfected with pCDH Flag cGAS-N or pCDH Flag cGAS-C plasmid for 36 h. The resultant cell lysates were mixed with OA at the noted pH for 1 h. After incubation with Pronase for 30 min, the mixtures were subjected to immunoblotting with the anti-Flag antibody. Input: the cell

`

lysates treated without Pronase; Digested: the cell lysates treated with Pronase. OA: oleic acid. *n* = 3.

K) Schematic showing the 10 K and 17 R residues in cGAS-N (amino acid 1–160).

L) Coomassie staining of purified human KR–A cGAS-N protein. KR–A cGAS-N mutant was generated by replacing K and R residues with alanine (A). kD: kilodalton.

`

Figure S3

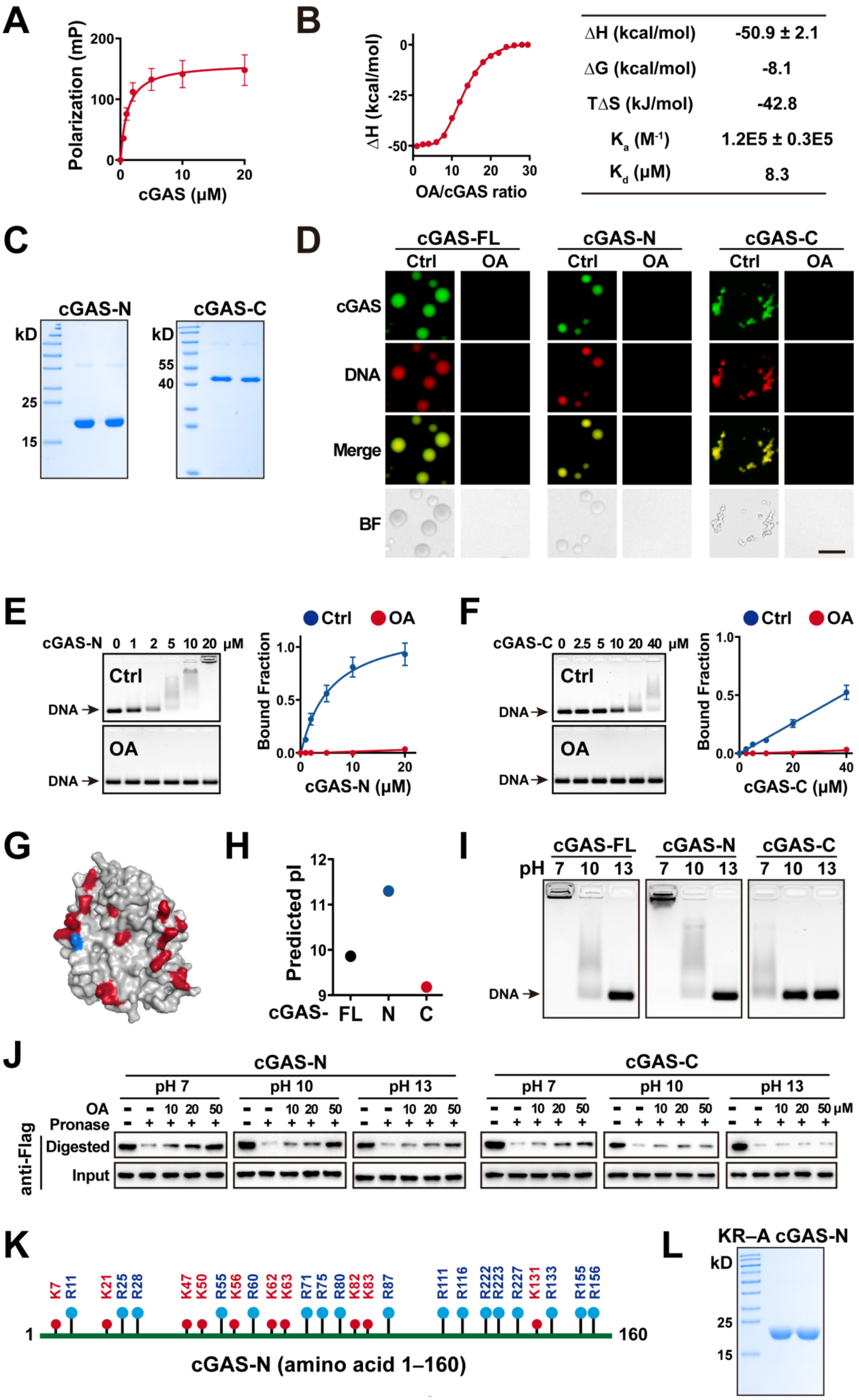
A
Polarization (mP)
cGAS (μM)
B
ΔH (kcal/mol)
OA/cGAS ratio
ΔH (kcal/mol) -50.9 ± 2.1
ΔG (kcal/mol) -8.1
TΔS (kJ/mol) -42.8
Ka (M-1) 1.2E5 ± 0.3E5
Kd (μM) 8.3
C
cGAS-N
cGAS-C
kD
D
cGAS-FL
cGAS-N
cGAS-C
Ctrl
OA
cGAS
DNA
Merge
BF
E
cGAS-N 0 1 2 5 10 20 μM
Ctrl
OA
DNA
Bound Fraction
cGAS-N (μM)
F
cGAS-C 0 2.5 5 10 20 40 μM
cGAS-C (μM)
G
H
Predicted pI
cGAS- FL N C
I
pH 7 10 13
J
Pronase
Digested
Input
anti-Flag
K
cGAS-N (amino acid 1–160)
K7 R11 K21 R25 R28 K47 K50 R55 K56 R60 K62 K63 R71 R75 R80 K82 K83 R87 R111 R116 R222 R223 R227 K131 R133 R155 R156
L
KR–A cGAS-N

Figure S4. FA dissolves the intracellular cGAS–DNA phase separation.

A) Representative IF images of the cGAS–DNA condensates formed via PS in mouse MEF cells treated with OA and then DNA transfection. MEF cells were treated with 1 mM OA for 1 h, and then transfected with 1 μg/mL FAM-ISD for 4 h, followed by IF analyses with anti-cGAS antibody. Unless specifically noted, distilled water was used as control (Ctrl). Red: anti-cGAS antibody; Green: FAM-labeled ISD DNA; Blue: DAPI. Scale bar = 10 μm. *n* = 3.

B) Representative IF images of the cGAS–DNA condensates in human U2OS cells treated with DNA transfection and then OA. U2OS cells were transfected with 1 μg/mL FAM-labeled ISD for 0.5 h. Then, the cells were treated with 1 mM OA (OA: BSA = 5: 1) for 4 h, and subjected to IF analyses with anti-cGAS antibody. Distilled water was used as control (Ctrl). Green: FAM-labeled ISD; Red: anti-cGAS antibody; Blue: DAPI. Scale bar = 10 μm. *n* = 3.

C) Representative IF images of the cGAS–DNA condensates in human U2OS cells treated with IR and then OA. U2OS cells were treated with 8 Gy IR. After 1 day, the cells were treated with 1 mM OA for 4 h, and then subjected to IF analyses with anti-cGAS and anti-dsDNA antibodies as noted. Green: anti-dsDNA antibody; Red: anti-cGAS antibody; Blue: DAPI. Scale bar = 10 μm. *n* = 3.

D) Representative IF images of the stress granules in the cells treated with OA. MEF cells were treated with 0.25 mM arsenic acid (As) and/or 1 mM OA for 4 h, followed by IF analyses with anti-G3BP stress granule assembly factor 1 (G3BP1) antibody. As: arsenic acid. Green: anti-G3BP1 antibody; Blue: DAPI. Scale bar = 10 μm. *n* = 3.

E) Immunoblotting of the lysates and streptavidin immunoprecipitates of the cytosolic fraction of the noted cells in the absence or presence of OA and biotin-labeled ISD. MEF and U2OS cytosolic lysates were mixed with 10 nM biotin-labeled ISD and/or 1 mM OA for 30 min. Then, the mixtures were incubated with streptavidin-coupled Dynabead at 4°C overnight, followed by the immunoblotting analyses with the appropriate antibodies. Input: the cell lysates treated without streptavidin-coupled Dynabead; IP: the streptavidin immunoprecipitates. Biotin-ISD: biotin-labeled oligo immune stimulatory DNA. OA: oleic acid. *n* = 3.

Figure S4

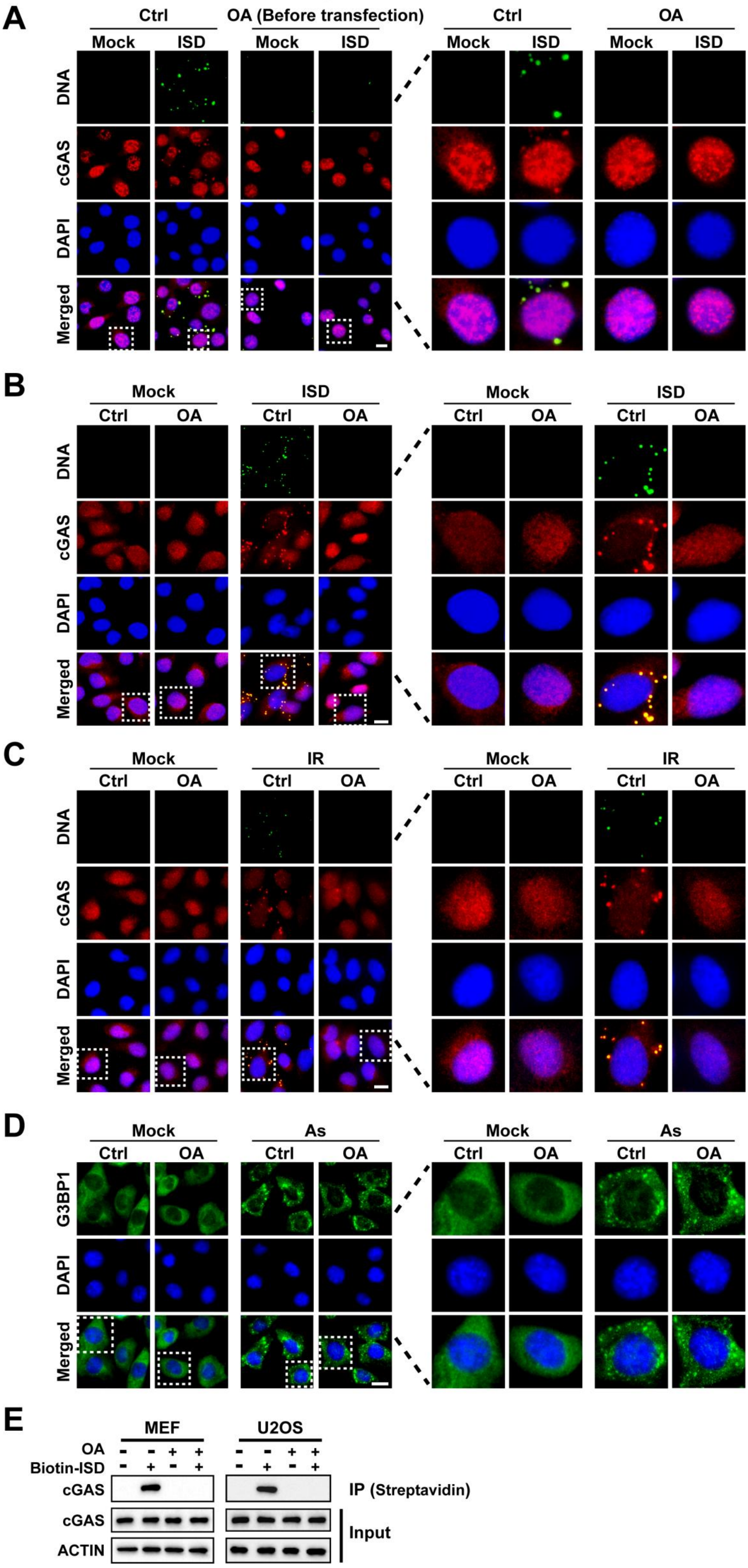

A
Ctrl
OA (Before transfection)
Ctrl
OA
Mock
ISD
DNA
cGAS
DAPI
Merged
B
Mock
ISD
Ctrl
OA
DNA
cGAS
DAPI
Merged
C
Mock
IR
Ctrl
OA
DNA
cGAS
DAPI
Merged
D
Mock
As
Ctrl
OA
G3BP1
DAPI
Merged
E
MEF
U2OS
OA
Biotin-ISD
cGAS
ACTIN
IP (Streptavidin)
Input

Figure S5. FA inhibits the cellular cGAS activation.

A) mRNA levels of the noted genes in the cells treated as described in Figure 5C. Unless specifically noted, Mean ± standard deviation (Mean ± SD) was used in this study. Unless specifically noted, *p* values were calculated by unpaired two-tailed Student's *t*-test, and *p* values < 0.05 were considered statistically significant (**p* < 0.05, ***p* < 0.01, ****p* < 0.001). *n* = 3.

B) mRNA levels of the noted genes in the cells treated as described in Figure 5G. *n* = 3.

C) mRNA levels of the noted genes in the cells treated as described in Figure 5I. Distilled water was used as mock of HSV infection. *n* =3.

D) Ifnb mRNA and cGAMP levels in the THP-1 cells treated with transfection and then OA. THP-1 cells were treated with 15 μM of cGAS inhibitor (cGASi, G150) for 1 h, and then 1 μg/mL of ISD or poly (I:C) (pIC) dsRNA transfection for 0.5 h. The resultant cells were treated with 1 mM OA for 8 h. Ifnb mRNA and cGAMP levels in the cells were analyzed by qRT-PCR and MS, respectively. pIC: poly (I:C) dsRNA. cGASi: cGAS inhibitor. *n* = 6.

E) IFN-β protein secreted from the THP-1 cells treated with transfection and then OA. THP-1 cells were treated with 15 μM cGASi for 1 h, and then 1 μg/mL of ISD or pIC dsRNA transfection for 0.5 h. The resultant cells were treated with 1 mM OA for 12 h. Then, Elisa was used to assess the secreted IFN-β protein. *n* = 3.

F) Ifnb mRNA and cGAMP levels in the THP-1 cells treated with OA and then transfection. THP-1 cells were treated with 15 μM cGASi and 1 mM OA for 1 h. Then, the cells were transfected with 1 μg/mL ISD or pIC dsRNA for 8 h. *n* = 6.

G) IFN-β protein secreted from the THP-1 cells treated with OA and then transfection. THP-1 cells were treated with 15 μM cGASi and 1 mM OA for 1 h. Then, the cells were transfected with 1 μg/mL ISD or pIC dsRNA for 12 h. *n* = 3.

H) Ifnb mRNA and cGAMP levels in the THP-1 cells treated with IR and then OA. The THP-1 cells were treated with 8 Gy of IR for 1 h. Then, the cells were treated with 15 μM cGASi and/or 1 mM OA for 24 h. *n* = 6.

I) IFN-β protein secreted from the cells described in (H). *n* = 3.

`

J) Ifnb mRNA and cGAMP levels in the THP-1 cells treated with OA and/or herpes simplex virus-1 (HSV) infection. THP-1 cells were treated with 15 μM cGASi and 1 mM OA for 1 h. Then, the cells were infected with 5 MOI of HSV for 8 h. n = 6.

K) IFN-β protein secreted from the cells treated with OA and then HSV infection. The THP-1 cells were treated with 15 μM cGASi and 1 mM OA for 1 h. Then, the cells were infected with 5 MOI of HSV for 12 h. n = 3.

`

Figure S5

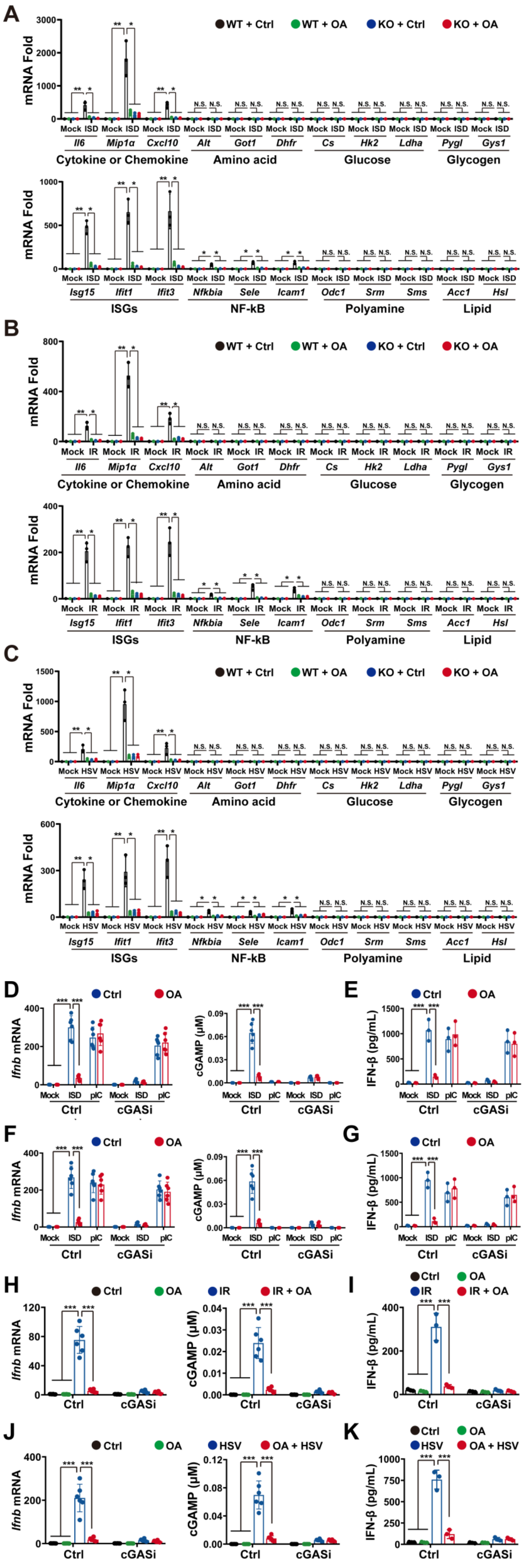
A
WT + Ctrl
WT + OA
KO + Ctrl
KO + OA
mRNA Fold
Mock ISD
Il6
Mip1α
Cxcl10
Alt
Got1
Dhfr
Cs
Hk2
Ldha
Pygl
Gys1
Cytokine or Chemokine
Amino acid
Glucose
Glycogen
Isg15
Ifit1
Ifit3
Nfkbia
Sele
Icam1
Odc1
Srm
Sms
Acc1
Hsl
ISGs
NF-kB
Polyamine
Lipid
B
Mock IR
C
Mock HSV
D
Ctrl
OA
Ifnb mRNA
cGAMP (μM)
Mock
ISD
pIC
cGASi
E
IFN-β (pg/mL)
F
G
H
IR
IR + OA
I
J
HSV
OA + HSV
K

Figure S6. FAs reduce cGAS-mediated anti-virus immunity.

A) ELISA analyses of IFN-α protein levels in the sera described in Figure 6B. Unless specifically noted, Mean ± standard deviation (Mean ± SD) was used in this study. Unless specifically noted, p values were calculated by unpaired two-tailed Student's t-test, and p values < 0.05 were considered statistically significant (*$p < 0.05$, **$p < 0.01$, ***$p < 0.001$). $n = 6$ mice.

B) ELISA analyses of IFN-α and IFN-β protein levels in the sera from the mice described in (A) at noted time points post infection. $n = 6$ mice.

C) mRNA levels of the noted genes in the peritoneal lavage cells from the mice treated with OA injection and then HSV infection. cGAS WT and KO mice were IP injected with 20 mg/mouse OA. After 2 h, the mice were infected by 1×10^7 pfu HSV. The peritoneal lavage cells were collected at 4 h post the infection. n = 3 mice.

D) Absolute (left) and relative (right) free fatty acid (FFA) levels in the guts (ilea) from the mice fed with standard rodent chow (Ctrl) or a HFD (60 kcal% fat diet) ad libitum for the noted times. The FFA level in the WT mice fed with standard rodent chow (Ctrl) was set to 1. $n = 6$ mice.

E) ELISA analyses of IFN-α protein levels in the sera described in Figure 6G. $n = 6$ mice.

`

Figure S6

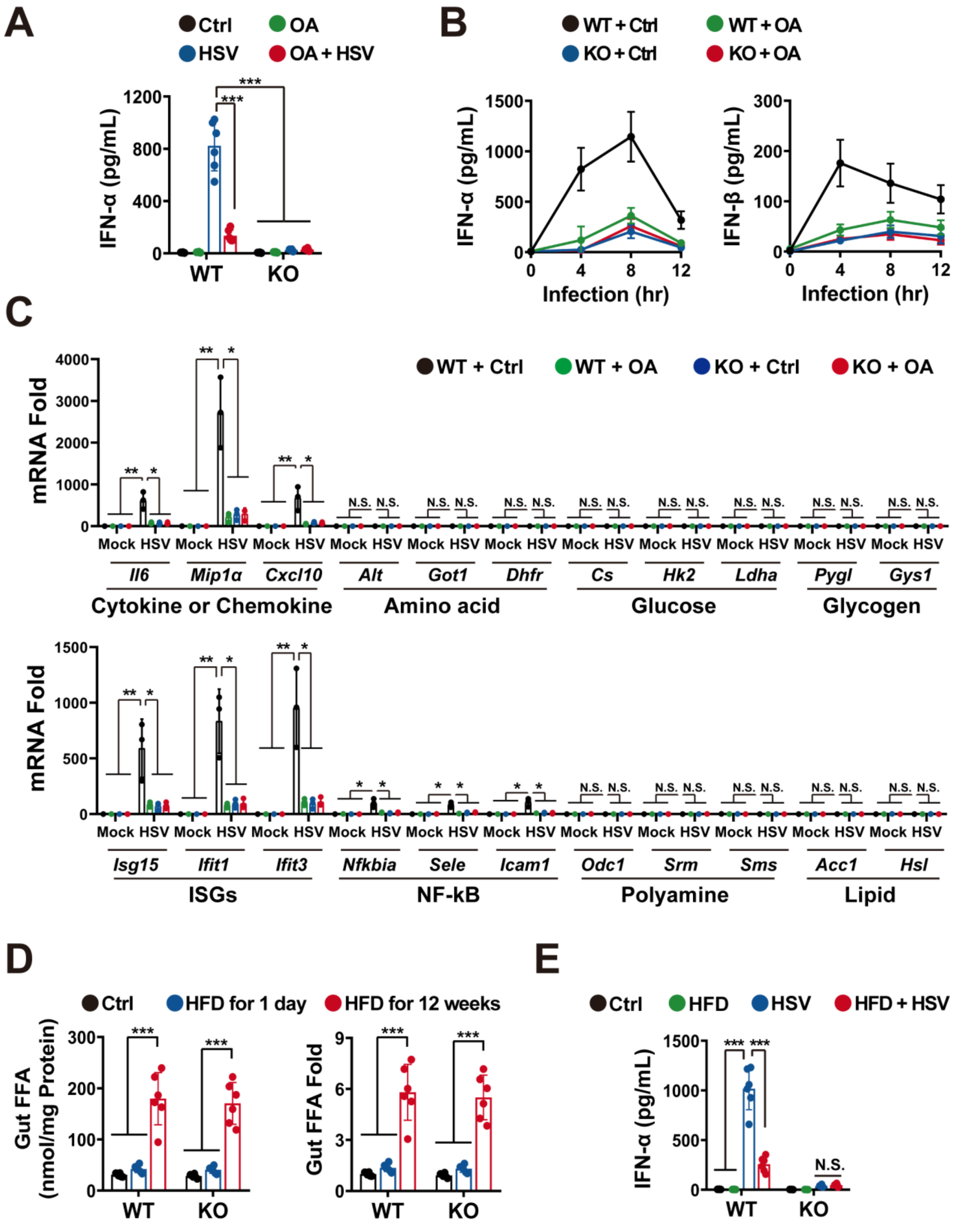
A
Ctrl
OA
HSV
OA + HSV
IFN-α (pg/mL)
WT
KO
B
WT + Ctrl
WT + OA
KO + Ctrl
KO + OA
IFN-α (pg/mL)
IFN-β (pg/mL)
Infection (hr)
C
mRNA Fold
Mock HSV
Il6
Mip1α
Cxcl10
Alt
Got1
Dhfr
Cs
Hk2
Ldha
Pygl
Gys1
Cytokine or Chemokine
Amino acid
Glucose
Glycogen
Isg15
Ifit1
Ifit3
Nfkbia
Sele
Icam1
Odc1
Srm
Sms
Acc1
Hsl
ISGs
NF-kB
Polyamine
Lipid
N.S.
D
Ctrl
HFD for 1 day
HFD for 12 weeks
Gut FFA (nmol/mg Protein)
Gut FFA Fold
E
Ctrl
HFD
HSV
HFD + HSV
IFN-α (pg/mL)

Figure S7. FAs attenuate cGAS-mediated anti-cancer immunity.

A) Relative serum FFA levels in the mice described in Figure 7A. Unless specifically noted, Mean ± standard deviation (Mean ± SD) was used in this study. Unless specifically noted, p values were calculated by unpaired two-tailed Student's t-test, and p values < 0.05 were considered statistically significant (*$p < 0.05$, **$p < 0.01$, ***$p < 0.001$). $n = 6$ mice.

B) Absolute FFA levels in the tumors described in Figure 7A. $n = 6$ mice.

C) mRNA levels of the noted genes in the tumors treated as described in Figure 7A. cGAS WT and KO mice were injected subcutaneously with 1×10^7 MC38 cells into the flank. After 10 days, the mice were treated with IP injection of OA at 20 mg/mouse and three focal fractions of 8 Gy IR on three consecutive days. The mRNA levels of the noted genes in the tumors were analyzed at 12 h post the third treatment of IR and OA. The saline was used as control. $n = 3$ mice.

D) The relative serum FFA levels in the mice described in Figure 7C. $n = 6$ mice.

E and F) The volumes of the tumors described in Figure 7D are presented to show the impact of HFD on the cGAS-dampened tumor growth in the context of IR. $n = 8$ mice.

G) Representative IF images of CD8+ cells in the tumors described in Figure 7E. Red: anti-CD8 antibody; Blue: DAPI. Scale bar = 100 µm.

H) B16 tumor volumes in the mice treated with HFD and IR. The mice pretreated with or without HFD for 12 weeks were treated with subcutaneously injection of 1×10^7 B16 cells. After 5 days, three focal fractions of 8 Gy of IR were used to irradiate the mice on three consecutive days. For HFD groups, the mice were consistently treated with HFD during the experiment. $n = 6$ mice.

I) CD8+ cell levels in the tumors described in (H). $n = 6$ mice.

J) LL/2 tumor volumes in the mice treated with HFD and IR. The mice pretreated with or without HFD for 12 weeks were treated with subcutaneously injection of 1×10^7 LL/2 cells. After 5 days, three focal fractions of 8 Gy of IR were used to irradiate the mice on three consecutive days. For HFD groups, the mice were consistently treated with HFD during the experiment. $n = 6$ mice.

K) CD8+ cell levels in the tumors described in (J). $n = 6$ mice.

`

L) Cell population scores in the tumors from control (BMI < 35, *n* = 243) and severely obese (BMI > 35, *n* = 34) COAD patients. The intratumor cell population scores were calculated by MCPcounter using default parameters. Mean + SD.

`

Figure S7

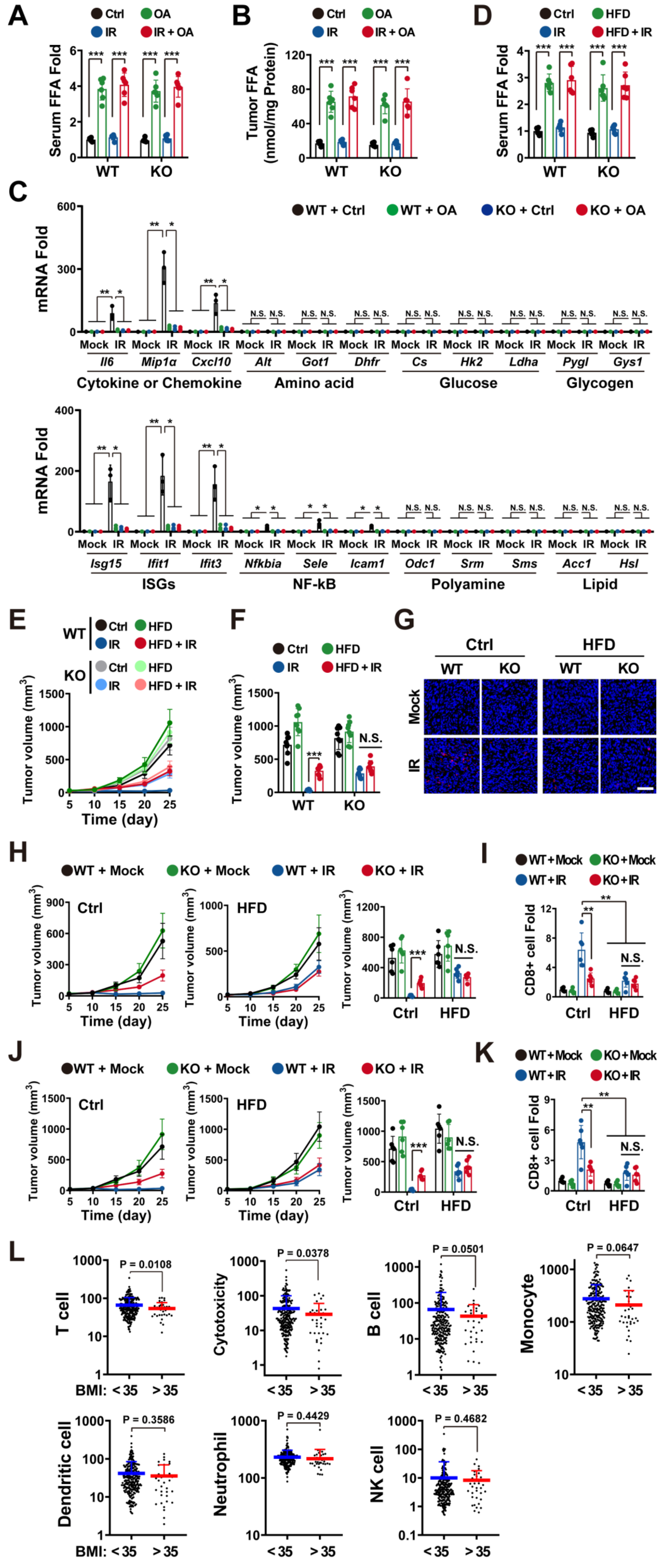

A
Ctrl
OA
IR
IR + OA
Serum FFA Fold
WT
KO
B
Tumor FFA (nmol/mg Protein)
D
HFD
HFD + IR
C
WT + Ctrl
WT + OA
KO + Ctrl
KO + OA
mRNA Fold
Mock
IR
Il6
Mip1α
Cxcl10
Alt
Got1
Dhfr
Cs
Hk2
Ldha
Pygl
Gys1
Cytokine or Chemokine
Amino acid
Glucose
Glycogen
Isg15
Ifit1
Ifit3
Nfkbia
Sele
Icam1
Odc1
Srm
Sms
Acc1
Hsl
ISGs
NF-kB
Polyamine
Lipid
N.S.
E
Tumor volume (mm3)
Time (day)
F
G
Mock
H
WT + Mock
KO + Mock
WT + IR
KO + IR
I
CD8+ cell Fold
J
K
L
T cell
Cytotoxicity
B cell
Monocyte
Dendritic cell
Neutrophil
NK cell
BMI:
< 35
> 35
P = 0.0108
P = 0.0378
P = 0.0501
P = 0.0647
P = 0.3586
P = 0.4429
P = 0.4682

Table S1. Turbidities of metabolite mixtures described in Figure 1C. The turbidity of Ctrl (distilled water) was taken as 1. Unless specifically noted, Mean ± standard deviation (Mean ± SD) was used in this study.

Molecules	replicate 1	replicate 2	replicate 3	Mean
Control (distilled water)	0.956	1.136	0.913	1.00
α-Linolenic acid (ALA)	0.122	0.095	0.005	0.07
Linoleic acid (LA)	0.051	0.003	0.099	0.05
Oleic acid (OA)	0.032	0.046	0.005	0.03
1,6-hexanediol (Hex)	0.831	0.948	1.076	0.95
Arachidonic acid (AA)	0.421	0.33	0.576	0.44
EicosapentaenoicAcid (EPA)	0.441	0.652	0.526	0.54
Docosahexaenoic Acid (DHA)	0.389	0.563	0.425	0.46
Palmitic acid (PA)	0.492	0.578	0.632	0.57
Dihomo-γ-linolenic Acid (20:3, n-6)	0.754	0.658	0.801	0.74
Spermine	1.15	1.236	1.065	1.15
Spermidine	0.989	1.068	1.175	1.08
Arginine (Arg)	0.935	0.982	1.054	0.99
Putrescine	1.021	0.929	0.974	0.97
Ornithine	0.869	1.134	0.957	0.99
cadaverine	1.103	0.921	0.984	1.00
itaconate	0.954	1.14	1.032	1.04
creatine	0.936	1.105	0.979	1.01

`

Lysine (Lys)	0.958	0.983	1.152	1.03
Histidine (His)	0.935	0.972	1.135	1.01
N1-Acetyl-spermidine	0.869	1.032	0.953	0.95
NADPH	0.962	1.032	0.906	0.97
NADH	0.976	0.889	1.068	0.98
Nicotinamide adenine dinucleotide phosphate	0.856	0.964	1.113	0.98
Fe2+	0.934	0.978	1.056	0.99
Mg2+	1.128	1.056	0.955	1.05
Zn2+	0.961	1.105	1.053	1.04
Nicotinamide adenine dinucleotide (NAD)	0.964	1.087	0.896	0.98
Vitamin C (Vc)	0.979	1.156	0.883	1.01
Adenosine triphosphate (ATP)	1.105	0.961	0.928	1.00
α-Ketoglutarate (αKG)	0.948	1.149	0.873	0.99
Dehydroascorbate (DHA)	1.032	0.937	0.895	0.95
Dihydrofolate (DHF)	1.025	0.833	0.965	0.94
Flavin mononucleotide (FMN)	0.876	0.977	1.103	0.99
Adenosine diphosphate (ADP)	0.855	0.938	1.061	0.95
N-Acetyl-L-cysteine (NAC)	0.869	0.926	1.097	0.96
Tetrahydrofolate (THF)	0.948	0.893	1.106	0.98
Methionine (Met)	0.915	0.982	1.115	1.00
methyltetrahydrofolate (MTHF)	1.106	0.861	0.931	0.97

Homocysteine	0.946	1.104	1.049	1.03
Nicotinamide (NAM)	1.011	0.925	1.038	0.99
Alanine (Ala)	1.064	0.869	1.147	1.03
Isoleucine (Ile)	0.908	0.983	1.091	0.99
Nicotinamide mononucleotide (NMN)	0.954	0.913	1.108	0.99
Acetoacetic acid (ACAC)	1.115	1.053	0.926	1.03
S-Adenosyl-L-homocysteine (SAH)	0.865	0.972	1.084	0.97
L-2-hydroxyglutarate(L-2HG)	0.932	0.876	1.152	0.99
Glutamate (Glu)	0.982	1.103	0.887	0.99
α--ketoisocaproic acid (KIC)	0.971	0.891	1.121	0.99
glutathione, reduced (GSH)	0.964	1.141	0.865	0.99
Aconitate	0.935	1.055	1.107	1.03
Acetyl phosphate	1.102	1.035	0.965	1.03
Mannose	0.859	1.154	1.068	1.03
S-adenosylmethionine (SAM)	1.032	0.926	0.978	0.98
phenylalanine (Phe)	1.056	0.954	1.063	1.02
Adenosine monophosphate (AMP)	0.878	0.915	0.798	0.86
Pyrophosphate	0.956	1.028	1.107	1.03
Proline (Pro)	0.948	0.869	1.147	0.99
Citrulline	1.065	0.914	0.959	0.98
Glyceraldehyde	0.876	1.109	0.951	0.98
Acetyl-CoA	0.928	0.891	1.169	1.00

`

Serine (Ser)	1.057	0.943	1.107	1.04
Tryptophan (Trp)	1.036	0.875	1.141	1.02
Valine (Val)	0.951	1.025	1.163	1.05
D-2-hydroxyglutarate (D-2HG)	1.042	0.867	0.938	0.95
Adonitol	0.875	0.926	1.108	0.97
Succinate	0.936	0.887	1.065	0.96
Asparagine (Asn)	1.065	0.957	0.849	0.96
α-ketoisovaleric acid (KIV)	0.922	0.876	1.105	0.97
glucose-6-phosphate (G6P)	1.157	1.063	0.948	1.06
Cystine	1.171	1.045	0.981	1.07
Urea	0.867	0.965	0.795	0.88
Pyruvate	1.012	0.869	0.957	0.95
Isocitrate	0.991	1.113	1.192	1.10
Glycerophosphorylcholine (GPC)	0.892	0.748	1.065	0.90
Mannitol	0.932	0.885	1.146	0.99
Glutamine (Gln)	0.986	0.893	1.055	0.98
Citrate	0.914	1.154	1.046	1.04
Glycerol 2-phosphate	1.028	1.176	0.958	1.05
Acetic acid	1.061	0.973	1.108	1.05
Sorbitol	0.891	0.95	0.993	0.94
Histamine	1.152	0.829	1.071	1.02
Leucine (Leu)	0.846	0.925	1.103	0.96

`

glutathione, oxidized (GSSG)	0.956	0.806	1.108	0.96
Fumarate	0.798	0.835	0.997	0.88
Sucrose	0.867	0.926	1.125	0.97
Oxaloacetate (OAA)	0.793	0.887	0.965	0.88
Glycerol	0.925	0.908	1.068	0.97
Phosphocreatine	0.865	0.934	1.035	0.94
Mannose-6-phosphate (M6P)	0.875	0.986	1.068	0.98
Tyrosine	1.025	1.133	0.934	1.03
fructose bisphosphate (FBP)	0.862	0.935	1.135	0.98
D-Glucose (Glc)	1.065	0.789	0.954	0.94
Laurate (12:0)	0.738	0.806	0.905	0.82
3-Hydroxybutyrate (BHBA)	1.036	0.951	0.923	0.97
Threonine (Thr)	0.879	0.966	1.062	0.97
Creatinine	0.847	1.054	0.894	0.93
Glucose 1-phosphate	0.953	0.876	1.058	0.96
Leucine (Leu)	1.087	0.965	1.002	1.02
2-Aminobutyrate	0.961	0.857	1.058	0.96
Docosahexaenoate (DHA; 22:6n3)	0.518	0.705	0.624	0.62
Xylose	0.965	1.079	0.869	0.97
Leucine (Leu)	1.025	0.087	0.868	0.66
Lactose	1.061	1.011	0.925	1.00
4-hydroxybutyrate (4-HBT)	0.854	0.936	1.054	0.95

`

5-Methylthioadenosine (MTA)	0.875	0.904	1.147	0.98
Phosphoenolpyruvate	1.052	0.834	0.962	0.95
D-ribose	0.925	0.876	1.025	0.94
5-Oxoproline	1.054	0.854	0.954	0.95
Stearate (18:0)	0.678	0.725	0.581	0.66
Pyridoxine	0.879	0.912	0.766	0.85
Mannitol	1.056	0.875	0.922	0.95
1,3-diphosphoglycerate(1,3-BPG)	0.936	1.054	0.826	0.94
Malate	0.865	0.911	1.125	0.97
Lactate	1.003	0.867	0.935	0.94
fructose 6-phosphate (F6P)	0.954	1.025	1.061	1.01
3-Phosphoglycerate (3-PG)	0.896	0.948	1.105	0.98
α-Keto-β-methylvaleric acid (KMV)	0.971	0.924	1.012	0.97
Alpha-tocopherol	0.902	0.984	1.033	0.97
Cholate	0.905	0.845	1.123	0.96
5-Hydroxytryptophan (5-HTP)	0.807	0.865	0.947	0.87
Agmatine	0.931	0.869	1.124	0.97
Ribose 5-phosphate	0.928	1.042	1.027	1.00
Ribulose	0.936	0.857	0.921	0.90
Butyrylcarnitine	1.025	0.922	0.893	0.95
2,3-Diphosphoglycerate(2,3-BPG)	0.925	0.858	1.092	0.96
Glyceraldehyde 3-phosphate (G3P)	0.985	0.842	1.063	0.96

`

2-Phosphoglycerate (2-PG)	0.932	0.895	1.036	0.95
Galactose	0.875	0.932	0.987	0.93
Glycerol 3-phosphate	1.021	0.934	0.851	0.94
Xylulose	1.032	1.092	0.806	0.98
Xylulose 5-phosphate	0.906	0.981	1.065	0.98
Erythrose	0.927	1.154	0.895	0.99
Erythrose 4-phosphate	0.897	1.031	0.971	0.97
Fucose	1.056	1.126	0.869	1.02

`

Table S2. Bioinformatic data for colorectal adenocarcinoma patients from The Cancer Genome Atlas (TCGA) (n = 277).

Patient_ID	BMI	CD8 Expression	T cell	T CD8+ cell	Cytotoxicity	NK cell	B cell	Monocyte	Myeloid dendritic cell	Neutrophil	Endothelial cell
TCGA-WS-AB45	14.72	-0.61	54.02	23.33	84.38	15.14	18.76	1220.37	108.78	705.16	1707.86
TCGA-DY-A1H8	15.63	-0.41	59.88	90.68	19.30	0.71	3.33	115.23	7.10	198.99	182.37
TCGA-DY-A1DD	17.26	-0.02	40.57	125.71	13.61	1.57	4.51	121.92	16.63	242.34	196.83
TCGA-EI-6507	17.43	1.87	72.62	81.50	119.01	4.82	28.80	483.32	20.44	396.77	349.73
TCGA-AF-6655	17.78	-1.06	67.79	28.86	17.25	3.04	9.27	186.76	24.22	178.04	446.62
TCGA-F4-6569	18.52	0.14	66.53	55.90	71.57	5.81	67.05	514.72	50.72	258.47	696.88
TCGA-DM-A282	18.80	-0.77	26.71	61.03	4.92	1.28	5.71	145.30	15.62	176.92	242.45
TCGA-CA-5255	18.93	-1.12	30.36	7.77	11.81	2.64	22.54	115.86	17.98	207.33	172.36
TCGA-CM-6166	19.22	-0.90	60.82	40.12	6.76	1.27	6.66	85.12	16.89	142.75	214.24
TCGA-NH-A5IV	19.70	0.83	70.34	78.62	144.97	43.29	30.81	438.22	31.53	261.40	359.14
TCGA-EI-6885	19.71	-0.31	74.62	60.99	31.10	3.48	17.28	441.17	57.74	307.45	530.67
TCGA-T9-A92H	19.72	-1.09	39.00	5.05	13.04	1.10	9.15	68.45	10.94	187.18	175.66
TCGA-F5-6814	19.92	1.30	69.02	98.72	96.42	106.05	14.47	267.38	47.09	232.65	322.52
TCGA-G4-6315	20.07	-1.15	27.91	12.85	10.43	3.92	85.99	50.90	7.10	237.63	408.60
TCGA-DM-A1D4	20.18	-0.90	48.28	22.01	20.05	37.25	8.41	60.92	11.50	159.80	185.59
TCGA-AD-6899	20.24	0.04	109.70	59.41	52.80	2.67	85.69	521.88	81.75	189.49	536.43
TCGA-CA-5256	20.31	0.05	50.30	69.74	30.54	1.25	16.44	116.01	55.79	123.00	161.81
TCGA-F4-6463	20.48	1.56	65.54	254.00	43.06	15.94	32.13	213.50	31.00	238.33	541.21

`

TCGA-CA-6717	20.55	0.62	99.99	84.20	73.39	9.13	316.75	537.53	157.41	234.43	564.54
TCGA-CA-6719	20.62	-0.04	42.65	79.28	28.45	1.68	31.12	385.52	33.29	249.14	507.04
TCGA-A6-6782	20.64	0.43	60.31	72.31	65.86	5.61	63.60	353.68	56.61	244.65	583.23
TCGA-F4-6807	20.69	1.98	107.14	77.04	109.25	4.72	129.01	484.19	116.03	248.98	584.13
TCGA-G4-6588	20.69	-0.75	40.35	36.55	38.77	5.45	7.51	200.01	13.42	269.50	241.36
TCGA-CA-6716	20.80	-1.37	89.26	5.09	2.93	1.11	15.96	125.90	18.75	212.52	295.25
TCGA-DC-6155	20.81	-1.16	76.76	28.90	32.43	4.64	23.91	119.71	27.24	125.12	469.53
TCGA-D5-6533	20.96	-1.03	37.46	21.89	12.27	1.08	24.34	181.68	21.71	161.48	382.36
TCGA-F4-6459	20.96	0.99	35.88	205.47	21.79	6.89	40.42	316.65	34.69	222.58	509.68
TCGA-EI-6512	21.11	-1.09	37.47	16.93	21.46	3.31	13.35	252.46	25.71	298.45	280.67
TCGA-G4-6309	21.11	1.03	52.50	215.18	27.37	4.88	12.46	152.32	14.04	216.01	255.99
TCGA-AF-2690	21.29	2.44	154.11	166.15	99.77	5.03	217.05	1018.69	255.10	303.41	743.53
TCGA-F5-6465	21.45	-0.37	126.82	29.45	45.57	5.78	82.84	557.17	74.18	292.68	412.61
TCGA-AU-6004	21.48	-0.37	86.40	32.36	51.75	6.45	171.20	441.55	80.29	366.81	355.46
TCGA-D5-6531	21.51	0.28	112.61	45.95	72.64	1.98	19.02	347.52	23.79	228.55	219.73
TCGA-DC-5869	21.59	-0.79	33.63	47.27	11.30	0.60	14.29	163.74	49.69	103.75	309.53
TCGA-DM-A28C	21.60	-1.33	143.03	4.53	3.43	1.44	17.83	101.52	6.76	233.94	268.78
TCGA-A6-5664	21.63	10.34	104.51	1161.94	34.76	13.72	90.08	476.29	95.65	287.99	753.42
TCGA-CM-6161	21.67	0.56	114.41	126.34	33.96	12.10	25.16	293.18	62.96	211.88	266.54
TCGA-CM-4747	21.70	-1.15	30.89	14.43	7.35	1.44	17.02	107.19	15.68	157.03	333.51
TCGA-D5-6931	22.09	-0.47	75.74	32.74	28.46	8.19	86.30	314.18	38.17	244.81	336.93
TCGA-CA-6718	22.32	0.91	100.29	49.37	165.40	34.42	96.10	491.94	72.88	229.13	364.86

`

TCGA-AH-6903	22.34	-0.75	125.92	59.40	5.59	5.78	24.33	125.99	18.89	255.65	216.30
TCGA-CM-5349	22.35	-0.86	28.75	44.66	10.45	2.76	116.94	455.71	47.94	189.30	288.58
TCGA-AZ-6601	22.48	0.38	72.21	69.62	107.80	15.12	203.11	534.14	57.97	262.50	338.36
TCGA-DM-A1D0	22.49	-1.29	22.60	15.32	1.79	0.92	4.91	75.52	7.53	183.26	197.49
TCGA-EI-6883	22.53	-0.33	60.93	45.15	50.05	4.79	44.75	130.64	32.36	265.47	240.34
TCGA-DC-6154	22.63	-1.06	30.41	28.95	29.50	2.82	4.20	179.73	13.79	168.60	276.57
TCGA-A6-6780	22.71	1.05	122.73	76.62	148.59	101.94	19.09	810.32	52.99	168.00	219.83
TCGA-CA-6715	22.83	0.09	47.12	150.41	4.54	0.65	5.73	92.44	4.14	167.50	220.72
TCGA-F4-6854	22.84	0.10	65.63	66.02	42.52	2.26	32.49	195.04	30.23	226.01	403.45
TCGA-F4-6805	22.86	-0.62	60.61	35.94	27.57	7.30	64.65	436.48	116.80	237.50	474.34
TCGA-EF-5831	22.86	-1.07	30.32	12.33	21.20	1.66	21.10	181.15	23.33	152.57	307.22
TCGA-AY-A54L	22.99	-0.66	54.56	68.28	7.88	2.33	10.28	99.81	5.60	155.05	171.46
TCGA-D5-6539	23.14	-0.69	47.35	31.63	23.76	12.39	54.95	233.96	70.61	353.29	658.10
TCGA-CA-5254	23.19	-0.82	32.15	20.46	23.22	4.40	45.41	178.05	9.47	168.00	171.36
TCGA-G4-6311	23.22	-0.25	51.64	106.51	12.22	3.87	312.96	317.27	23.71	242.39	251.61
TCGA-DM-A1DA	23.34	-0.85	33.95	15.27	20.59	1.31	9.51	94.63	7.06	174.99	132.87
TCGA-F4-6570	23.34	2.77	101.47	85.65	163.76	9.99	46.78	746.98	42.43	265.60	405.30
TCGA-NH-A50V	23.46	-0.95	103.29	19.27	18.33	3.10	54.55	253.46	27.26	222.00	365.56
TCGA-DM-A285	23.51	-1.10	22.27	31.37	4.12	28.90	5.97	93.68	17.58	236.22	244.54
TCGA-AD-6895	23.53	1.04	76.21	86.90	143.80	10.20	27.22	165.23	63.47	262.75	300.78
TCGA-CM-6168	23.65	-0.76	70.51	14.32	41.47	4.58	98.38	577.84	95.56	288.77	509.28
TCGA-CM-6163	23.66	0.82	74.57	73.88	52.79	6.24	100.72	319.62	68.15	197.90	303.86

`

TCGA-F4-6806	23.66	-0.49	51.91	60.95	19.55	9.63	128.35	128.41	21.84	180.35	299.32
TCGA-A6-A567	23.77	-0.44	36.87	80.68	11.53	3.23	24.44	76.99	15.31	146.33	223.63
TCGA-A6-A56B	23.83	-1.36	30.75	8.32	3.06	3.72	4.76	152.03	21.40	205.31	318.13
TCGA-F4-6856	23.83	-0.33	50.37	36.34	51.64	2.01	18.26	92.05	24.46	169.83	298.92
TCGA-AD-6901	23.88	0.05	91.60	54.80	49.00	5.47	20.48	382.14	61.75	222.77	441.68
TCGA-A6-5656	23.91	-1.18	48.08	13.83	6.80	1.52	4.42	85.34	16.70	156.78	173.83
TCGA-A6-5661	23.91	-0.94	29.54	13.92	16.26	6.76	34.50	139.23	14.25	224.13	177.78
TCGA-CM-6678	23.94	-0.63	59.37	77.02	5.36	2.12	11.21	73.54	16.71	158.75	360.21
TCGA-5M-AATE	24.07	-0.39	40.45	84.44	8.86	2.15	24.03	121.02	20.59	186.27	239.36
TCGA-A6-6781	24.11	1.78	78.43	219.51	142.07	19.71	41.55	906.54	47.82	292.05	743.58
TCGA-DM-A0XD	24.22	-0.83	27.31	52.29	13.63	3.91	29.42	125.18	9.06	172.72	180.22
TCGA-D5-6927	24.26	1.52	91.99	123.54	85.28	16.64	22.78	406.63	35.43	278.85	317.46
TCGA-DM-A28A	24.34	-0.80	33.89	39.68	17.61	2.06	8.39	125.93	10.55	242.77	228.70
TCGA-F5-6861	24.39	-0.65	46.40	49.69	14.24	5.26	7.87	156.91	15.44	207.07	241.09
TCGA-DM-A1D7	24.49	-1.11	43.26	6.28	18.79	3.75	16.40	160.27	12.21	165.63	265.11
TCGA-G4-6302	24.51	0.18	52.84	95.48	37.26	7.54	38.60	413.34	83.86	369.14	986.85
TCGA-DC-6683	24.66	-1.25	28.03	8.24	8.05	0.71	45.19	163.89	22.14	180.87	210.20
TCGA-D5-6538	24.67	-0.41	24.43	87.99	10.39	1.07	4.49	82.99	7.27	298.27	277.20
TCGA-EI-6506	24.77	-0.42	62.84	50.27	31.81	8.34	73.19	298.31	42.85	299.33	564.60
TCGA-DM-A1HB	24.80	-1.37	28.04	1.81	7.20	2.67	8.87	102.01	13.46	199.88	202.08
TCGA-NH-A8F8	24.81	-1.19	54.90	6.18	13.99	2.60	28.78	234.54	15.50	189.52	334.45
TCGA-A6-5667	24.82	0.14	21.02	136.94	11.08	1.27	4.50	136.63	20.35	184.08	328.36

`

TCGA-D5-6540	24.86	0.42	40.44	100.86	72.20	8.56	9.15	194.79	31.99	268.39	316.92
TCGA-DM-A280	24.92	-1.28	18.36	15.88	8.27	14.40	2.48	93.28	6.01	162.47	193.26
TCGA-A6-6140	24.96	-0.22	55.29	62.69	38.52	3.05	16.11	86.94	13.41	250.82	294.62
TCGA-F4-6461	25.00	-0.86	68.44	27.30	17.03	3.34	722.42	171.34	32.76	267.37	557.06
TCGA-DM-A28F	25.06	-1.09	42.24	15.68	11.90	1.48	40.42	48.58	6.13	134.99	206.21
TCGA-AY-A8YK	25.16	-1.33	51.08	2.79	9.62	2.44	36.11	105.55	16.16	256.71	335.88
TCGA-DM-A1HA	25.22	-0.44	40.67	35.94	33.44	2.65	7.69	48.94	4.79	186.92	77.29
TCGA-D5-7000	25.30	-0.24	54.02	46.26	39.32	2.65	23.62	225.37	33.87	252.03	367.02
TCGA-DY-A0XA	25.32	-0.22	38.49	90.50	15.67	1.33	14.35	115.12	11.73	209.91	237.57
TCGA-EI-6511	25.34	2.23	113.83	127.22	112.12	4.55	117.01	496.80	35.89	217.29	455.07
TCGA-F4-6808	25.40	1.36	31.63	281.82	5.67	0.81	7.25	78.82	7.58	178.05	205.87
TCGA-F5-6812	25.46	0.01	74.66	70.76	34.67	3.15	152.08	292.94	78.74	202.52	474.66
TCGA-EI-6513	25.47	0.60	54.72	160.76	22.30	1.99	12.80	207.31	21.33	245.13	280.24
TCGA-DC-5337	25.49	-0.64	67.23	65.07	17.50	1.19	14.79	172.29	30.51	500.16	250.73
TCGA-A6-2686	25.56	4.22	173.99	182.35	347.72	7.07	77.28	627.23	203.12	338.89	356.99
TCGA-A6-5666	25.61	-1.30	95.66	6.11	3.93	0.51	10.83	57.78	6.42	150.55	199.36
TCGA-A6-4105	25.62	2.18	102.56	187.11	88.54	4.81	23.39	501.57	37.32	248.45	292.62
TCGA-F5-6702	25.69	-0.89	58.68	21.89	22.01	3.72	30.77	349.97	48.08	255.87	497.91
TCGA-D5-5538	25.71	0.06	67.80	86.47	34.87	4.88	63.68	622.04	63.55	222.10	297.87
TCGA-DM-A28K	25.76	-1.23	23.53	17.12	11.86	1.95	10.37	192.28	6.02	211.86	261.49
TCGA-G4-6307	25.77	-0.91	47.69	49.23	2.53	2.59	71.53	75.78	20.41	240.39	214.51
TCGA-QG-A5YX	25.78	-0.59	28.03	76.92	4.79	87.05	11.87	95.45	15.14	247.82	189.05

`

TCGA-A6-A566	25.82	-0.73	95.67	20.58	36.88	36.56	10.52	1243.79	72.21	226.39	1316.71
TCGA-G4-6627	25.83	0.83	34.82	147.40	20.63	1.17	13.41	86.55	7.18	178.48	138.39
TCGA-EI-6917	25.83	-0.33	97.22	60.58	82.58	11.68	231.36	251.57	69.62	277.00	577.35
TCGA-D5-6537	25.83	0.43	83.61	42.71	69.64	8.20	39.86	789.76	70.29	226.33	339.15
TCGA-AF-6136	25.83	-0.87	33.11	27.64	14.00	2.51	64.86	134.96	39.95	215.33	400.75
TCGA-F4-6460	25.86	-0.80	92.02	34.93	22.16	9.25	91.18	280.43	52.45	214.70	470.00
TCGA-EI-6509	25.88	-1.37	15.00	5.96	14.04	2.41	3.78	175.80	7.06	337.86	285.00
TCGA-AH-6643	25.91	-1.19	96.70	7.52	10.49	3.87	10.72	183.79	15.66	237.17	402.82
TCGA-AF-4110	26.03	2.19	92.29	212.30	65.21	8.01	258.20	313.08	70.93	351.12	491.08
TCGA-A6-6648	26.04	-0.16	29.99	86.91	16.85	10.48	31.80	110.07	26.18	274.07	374.13
TCGA-DM-A1D8	26.04	-0.41	73.76	32.38	35.40	3.63	26.64	86.32	22.53	181.65	158.38
TCGA-A6-5662	26.05	0.38	18.80	183.12	5.51	2.54	7.74	119.67	17.32	199.22	251.66
TCGA-EI-6508	26.11	-1.19	90.93	5.09	16.96	34.46	16.19	158.43	19.71	239.00	338.02
TCGA-CM-6164	26.22	0.86	56.36	166.85	37.08	2.78	17.75	128.60	33.67	190.11	403.21
TCGA-EI-6510	26.22	0.17	96.42	123.29	17.31	13.39	100.79	75.09	23.21	206.06	256.17
TCGA-D5-6535	26.37	-0.70	79.06	25.33	20.95	3.41	40.01	243.23	44.92	214.35	423.50
TCGA-A6-2684	26.38	-0.51	128.35	32.92	33.72	3.94	34.43	427.78	95.15	212.00	346.13
TCGA-A6-6138	26.39	1.65	98.08	62.05	144.09	7.87	69.92	539.92	73.28	210.93	519.80
TCGA-A6-2685	26.44	-0.72	70.25	26.52	20.16	6.35	96.30	602.57	98.80	313.74	474.68
TCGA-EI-6884	26.49	0.04	66.89	61.75	40.72	6.10	82.05	324.07	54.68	311.56	495.64
TCGA-AH-6897	26.52	0.30	22.01	168.74	7.03	0.66	11.72	68.80	5.98	140.46	213.14
TCGA-D5-6932	26.57	-0.95	62.11	93.53	7.49	0.93	39.28	150.72	12.57	184.82	229.64

`

TCGA-D5-5537	26.57	-0.40	93.15	19.85	25.76	2.76	7.73	218.92	19.04	192.53	375.52
TCGA-D5-6930	26.58	3.74	84.01	387.41	138.83	8.80	497.54	277.54	39.95	211.08	369.20
TCGA-CM-5344	26.67	-0.45	27.76	85.32	15.64	345.27	13.50	137.17	20.22	172.84	348.58
TCGA-AD-A5EJ	26.72	-1.10	32.65	4.39	60.80	12.43	16.51	144.98	48.34	201.98	262.27
TCGA-DM-A1D6	26.73	-0.86	15.87	54.50	3.84	0.53	36.04	61.51	3.84	151.31	190.03
TCGA-D5-6926	26.78	-0.77	45.83	23.86	37.52	4.37	31.85	355.59	26.33	244.06	425.40
TCGA-CM-4744	26.78	2.47	63.85	112.47	117.38	5.60	21.50	81.28	26.05	117.67	106.71
TCGA-CM-6675	26.85	-0.97	25.82	12.09	18.37	3.00	16.27	147.53	9.83	186.13	200.30
TCGA-D5-6530	26.87	0.18	96.84	53.02	78.38	25.58	55.31	412.98	44.10	249.91	249.62
TCGA-DM-A1DB	26.89	3.04	52.62	351.54	38.67	6.13	10.11	132.43	17.32	172.31	188.83
TCGA-F5-6464	26.90	1.02	77.00	68.60	63.59	5.39	29.87	643.22	104.03	241.44	584.82
TCGA-D5-6922	27.10	-0.50	27.71	86.21	40.46	2.54	4.31	107.58	20.20	86.49	147.07
TCGA-D5-5540	27.10	-0.13	52.30	40.32	32.18	7.34	14.09	346.27	34.42	246.53	364.18
TCGA-CM-6171	27.13	-0.86	37.76	16.65	22.88	4.61	9.00	246.45	22.40	521.16	307.61
TCGA-G5-6235	27.17	-0.40	29.30	66.46	18.89	6.66	72.95	76.12	10.74	505.35	252.67
TCGA-AD-6964	27.17	3.37	165.17	223.23	131.88	70.12	107.41	888.99	67.37	230.45	455.86
TCGA-EI-7002	27.17	0.28	49.04	159.30	9.65	1.58	15.97	266.88	34.39	178.17	405.64
TCGA-DC-4749	27.18	-0.45	32.08	95.05	7.30	3.81	12.88	86.14	13.53	159.03	192.04
TCGA-G4-6586	27.32	3.27	84.61	156.95	188.73	2.66	20.20	105.73	18.12	220.56	237.71
TCGA-DY-A1DE	27.34	-0.51	59.64	31.87	30.83	3.31	15.57	228.94	28.43	127.43	305.50
TCGA-G4-6322	27.44	-1.10	83.22	5.65	36.96	3.23	28.41	107.39	24.47	182.08	561.48
TCGA-DM-A28H	27.46	-0.67	41.81	69.57	4.12	1.19	13.59	87.06	6.64	143.43	187.93

`

TCGA-DC-6681	27.67	-0.75	96.59	40.95	11.92	96.42	18.36	174.81	31.11	215.86	442.63
TCGA-AD-A5EK	27.70	-0.84	49.95	48.28	10.84	1.48	19.68	124.63	17.93	237.06	255.07
TCGA-EI-6881	27.76	-1.21	49.85	3.76	12.30	1.75	23.64	120.29	20.25	247.97	262.64
TCGA-A6-2675	27.85	-0.95	53.91	10.79	43.93	10.99	43.96	526.89	51.03	278.32	396.59
TCGA-F5-6863	27.94	-0.74	35.05	68.03	3.56	1.77	4.09	95.17	20.37	213.71	330.61
TCGA-DC-6158	28.01	-0.42	94.98	19.98	60.37	3.86	30.25	462.86	87.71	223.78	386.93
TCGA-D5-5541	28.03	0.51	43.78	114.65	39.16	2.10	18.99	429.50	45.11	182.57	272.37
TCGA-D5-6541	28.08	0.61	52.34	59.10	65.63	3.47	41.16	626.68	57.69	203.57	279.72
TCGA-F5-6813	28.08	0.22	39.26	56.79	41.13	1.97	99.93	174.25	34.51	141.31	371.38
TCGA-EF-5830	28.08	-0.88	26.30	17.17	68.04	3.66	1.64	162.12	18.38	138.92	187.41
TCGA-DY-A1DG	28.08	-0.70	40.64	57.38	10.37	1.23	7.36	81.83	11.48	175.94	161.24
TCGA-DC-6156	28.11	5.48	102.02	275.14	142.21	2.99	31.47	775.53	61.43	245.59	614.07
TCGA-CM-6165	28.19	-0.64	39.00	30.03	20.52	2.58	26.71	317.85	63.81	214.34	429.29
TCGA-AD-6965	28.37	-0.59	53.57	42.36	17.64	2.26	33.51	132.67	14.67	192.87	295.12
TCGA-F4-6703	28.39	1.66	156.50	81.97	223.10	18.62	106.75	1328.51	130.84	357.22	750.21
TCGA-D5-6920	28.52	-0.64	129.13	36.22	28.18	93.36	59.69	250.90	30.31	525.72	407.53
TCGA-A6-6137	28.61	-1.01	92.83	14.77	12.40	21.86	58.04	261.81	71.26	259.31	498.67
TCGA-AF-A56N	28.65	-0.59	74.40	75.62	7.28	1.16	17.64	163.97	24.83	202.99	319.22
TCGA-AD-6888	28.67	-0.96	31.83	39.73	5.62	1.58	10.44	82.75	12.70	297.09	152.19
TCGA-G4-6293	28.67	0.12	112.07	40.83	50.86	5.97	84.44	211.71	31.92	223.97	353.99
TCGA-D5-6929	28.69	-0.46	108.67	43.15	26.06	2.73	149.17	245.38	17.26	150.96	290.28
TCGA-F4-6855	28.69	-1.14	30.67	11.13	21.88	3.85	12.52	356.17	35.22	170.23	468.37

`

TCGA-NH-A50T	28.70	-1.28	17.66	12.12	5.64	11.67	5.22	66.93	5.56	146.50	165.06
TCGA-AD-6963	28.73	1.54	87.30	118.29	72.61	2.66	17.42	203.50	32.36	232.95	232.34
TCGA-AH-6544	28.79	-1.31	13.24	9.56	3.14	1.67	71.25	43.89	7.26	184.01	169.14
TCGA-CM-6674	28.94	0.96	45.59	161.40	74.31	8.32	33.79	244.44	24.49	205.19	301.71
TCGA-F5-6571	29.03	-0.02	94.34	38.94	51.80	3.49	33.07	470.53	128.46	277.34	326.96
TCGA-AZ-4615	29.07	4.77	93.88	182.78	208.51	7.42	7.89	265.42	64.07	242.67	176.07
TCGA-AF-3911	29.07	-0.13	43.32	94.78	22.01	1.74	13.67	172.09	28.43	166.56	306.29
TCGA-QG-A5Z2	29.11	1.66	96.16	112.44	67.78	29.25	572.89	154.69	34.14	273.89	195.64
TCGA-QG-A5YW	29.19	-1.07	97.51	9.59	15.18	21.97	64.80	263.09	26.12	242.73	337.96
TCGA-A6-6649	29.36	0.03	66.51	69.52	33.37	6.90	106.31	335.35	96.31	402.73	742.65
TCGA-CM-5862	29.39	-0.69	33.75	74.51	9.79	2.61	1.39	57.70	17.12	140.65	336.99
TCGA-AF-A56L	29.39	-0.91	61.78	29.06	11.81	1.27	19.67	149.06	20.60	202.89	255.40
TCGA-CM-6167	29.40	-0.46	84.37	41.34	42.40	6.16	153.68	449.29	167.99	285.74	1137.02
TCGA-D5-5539	29.41	-0.87	33.45	34.76	16.16	5.70	51.50	278.16	48.01	257.77	397.30
TCGA-DM-A0XF	29.41	-1.13	50.96	21.35	30.57	2.18	5.06	163.16	15.14	141.09	130.51
TCGA-DM-A288	29.41	-1.36	23.40	8.96	2.32	0.84	5.39	47.76	3.70	174.68	188.33
TCGA-A6-6653	29.42	-1.16	24.94	12.11	33.66	4.16	6.18	212.01	46.32	287.22	317.64
TCGA-G4-6625	29.50	1.03	144.19	54.88	109.27	8.45	253.40	577.52	145.31	229.99	420.35
TCGA-NH-A6GB	29.55	-0.76	67.55	46.32	26.62	3.17	23.55	120.53	12.48	230.06	209.79
TCGA-A6-A565	29.64	2.67	198.63	177.82	91.71	13.77	618.53	382.84	68.84	344.30	995.13
TCGA-NH-A50U	29.67	-0.86	61.34	56.61	5.28	40.45	4.50	224.33	8.04	201.74	293.00
TCGA-EI-7004	29.75	-0.80	67.44	36.36	24.49	5.71	92.48	712.08	81.99	297.86	2039.71

`

TCGA-DM-A0X9	29.76	0.23	70.76	42.72	50.36	2.74	19.27	104.86	34.18	206.94	265.36
TCGA-A6-2682	29.78	-0.66	55.52	68.46	19.05	5.86	19.43	241.34	23.68	218.76	336.19
TCGA-G4-6323	30.06	-0.28	92.30	53.42	27.05	14.99	1194.47	102.43	32.41	189.98	331.85
TCGA-CM-4743	30.26	0.15	46.03	113.95	108.88	9.11	11.60	179.48	67.25	245.25	271.73
TCGA-D5-6532	30.39	-0.79	55.80	54.48	7.03	1.15	23.29	78.01	5.62	178.12	200.48
TCGA-CM-5860	30.40	-0.62	47.84	31.19	43.91	2.21	12.43	348.02	61.92	182.38	327.71
TCGA-DM-A28M	30.42	-0.57	34.65	87.64	12.67	6.95	13.41	44.16	6.76	128.34	169.93
TCGA-D5-6536	30.49	0.91	43.32	236.28	10.01	3.27	16.54	281.86	13.79	308.03	450.70
TCGA-A6-6141	30.62	1.58	127.41	116.87	70.91	25.30	160.39	198.24	74.55	198.39	312.40
TCGA-D5-6928	30.72	15.79	342.05	586.88	546.29	38.54	528.27	1212.49	160.14	451.33	519.03
TCGA-DY-A1DF	30.82	-1.45	150.33	1.30	21.44	3.16	6.93	65.97	12.93	246.14	241.42
TCGA-AM-5821	31.04	1.58	50.13	80.09	148.13	6.39	72.12	509.85	39.20	184.71	205.08
TCGA-DM-A28G	31.14	-0.30	46.92	83.03	13.92	22.94	20.18	160.07	21.56	157.16	308.60
TCGA-D5-6923	31.17	-1.21	43.21	10.14	16.43	3.84	7.16	228.13	37.36	246.14	526.55
TCGA-G5-6233	31.22	-1.14	63.24	7.62	10.40	3.75	26.77	219.28	20.09	198.92	514.10
TCGA-D5-6924	31.22	0.32	74.89	89.96	39.76	4.63	97.98	437.62	45.38	244.71	504.10
TCGA-AF-6672	31.45	0.41	71.24	167.53	16.99	5.81	106.81	101.17	20.40	222.51	369.09
TCGA-G4-6321	31.49	1.35	61.92	85.64	102.28	13.87	197.71	98.79	27.40	333.95	456.74
TCGA-F5-6864	31.64	0.15	60.15	113.55	31.63	22.76	27.02	291.07	51.55	216.86	481.52
TCGA-AD-6548	31.69	0.22	77.10	65.37	70.19	5.97	60.06	378.54	42.49	242.43	361.84
TCGA-CM-5864	31.79	-0.71	45.15	44.96	14.79	0.94	41.89	110.94	23.43	178.52	269.25
TCGA-G4-6628	31.86	5.50	148.63	208.13	279.08	26.02	50.84	523.34	106.22	235.66	386.28

`

TCGA-CM-6680	32.18	-0.36	128.37	52.83	33.03	8.98	41.01	320.11	41.15	252.18	387.72
TCGA-CM-5348	32.18	-0.95	56.41	19.31	21.42	2.41	25.82	629.84	74.64	395.63	383.85
TCGA-F5-6811	32.32	1.19	51.69	242.48	24.75	5.58	39.36	289.94	35.53	170.43	289.00
TCGA-D5-6534	32.53	0.83	168.09	86.43	68.10	6.58	129.82	1099.63	394.87	304.63	716.83
TCGA-DC-6160	32.54	-0.31	68.13	78.47	22.00	2.76	61.55	200.58	29.18	265.05	345.65
TCGA-A6-6654	32.54	0.59	107.82	66.99	75.97	7.91	59.99	917.25	137.00	248.07	671.43
TCGA-A6-5660	32.66	-1.15	26.97	14.18	9.08	1.61	9.18	249.26	29.38	163.40	275.83
TCGA-G5-6641	33.10	-1.26	27.75	8.26	16.46	3.25	15.12	47.48	9.83	196.17	245.89
TCGA-AF-2693	33.10	-0.78	48.81	16.01	19.66	2.70	66.65	208.84	35.57	286.66	404.62
TCGA-A6-6142	33.33	-0.23	38.81	120.34	9.68	1.68	7.30	291.51	18.46	273.29	469.54
TCGA-DM-A28E	33.33	-1.33	42.50	10.33	2.95	1.12	9.77	91.54	5.66	202.75	218.23
TCGA-A6-6651	33.47	0.83	98.66	105.12	50.68	4.08	404.46	862.08	145.66	311.73	887.12
TCGA-G4-6314	33.48	-0.84	45.72	36.99	15.33	1.61	22.91	235.17	48.78	221.93	526.99
TCGA-CM-6679	33.61	-0.88	68.81	19.34	18.72	1.04	144.68	245.35	51.93	209.81	396.24
TCGA-A6-5657	33.67	-0.20	93.71	41.43	34.25	3.08	764.84	166.19	42.95	234.77	454.74
TCGA-AU-3779	33.67	1.67	108.98	310.14	12.66	2.80	80.92	368.81	80.38	200.99	285.77
TCGA-DC-6157	33.89	-0.89	97.13	35.98	8.71	1.12	19.74	197.62	32.48	163.52	315.05
TCGA-CM-6172	34.03	-1.01	38.23	35.59	5.53	22.24	44.25	181.90	47.33	269.13	402.20
TCGA-EI-6882	34.09	0.04	39.49	95.48	44.61	12.63	45.74	213.02	21.15	306.16	391.51
TCGA-CM-5868	34.13	-0.84	51.89	53.05	8.81	2.20	10.08	112.18	14.48	139.31	224.06
TCGA-D5-6529	34.22	-0.03	123.27	53.84	44.89	2.99	213.36	455.46	56.33	300.48	365.90
TCGA-G4-6310	34.25	-0.83	15.50	60.63	2.67	1.03	2.99	56.34	16.77	190.47	263.20

`

TCGA-A6-A5ZU	34.29	-0.81	63.14	20.06	32.65	4.35	134.40	352.29	21.93	270.32	288.27
TCGA-CM-4751	34.34	0.88	106.65	54.39	77.77	8.46	108.55	255.47	116.09	185.65	412.88
TCGA-DC-4745	34.45	-1.11	52.40	14.60	13.03	1.60	13.86	131.06	36.95	120.41	300.29
TCGA-NH-A6GA	34.80	0.72	52.09	184.85	22.10	1.78	20.15	233.44	34.32	248.86	376.52
TCGA-SS-A7HO	34.96	0.59	68.91	196.26	7.97	0.82	2.23	137.82	6.88	223.91	371.32
TCGA-CM-6169	35.29	1.31	101.59	112.62	69.23	6.39	59.57	667.96	134.52	287.87	699.67
TCGA-RU-A8FL	35.38	-1.33	22.61	6.81	2.66	1.08	8.23	24.65	2.82	115.02	133.26
TCGA-AF-5654	35.48	-1.34	34.28	7.07	4.61	1.77	4.49	91.79	10.66	174.65	230.94
TCGA-CM-6677	35.68	-0.60	60.02	34.53	33.56	20.73	62.29	178.53	39.54	175.27	399.51
TCGA-A6-6650	35.82	-1.30	34.96	7.38	4.27	1.23	17.03	113.84	6.44	176.72	294.24
TCGA-A6-5659	36.14	-1.27	27.90	5.96	8.29	1.12	20.92	165.63	30.92	163.27	285.56
TCGA-G4-6317	36.31	-1.42	20.29	3.31	0.79	0.62	2.14	68.32	4.52	114.36	201.64
TCGA-AM-5820	36.44	-1.13	12.59	16.28	8.47	0.89	8.35	194.75	18.87	153.29	341.46
TCGA-QG-A5YV	36.50	-0.88	53.89	25.98	12.43	3.49	14.81	95.73	16.85	179.11	246.45
TCGA-CM-6676	36.73	-1.15	36.30	12.02	8.24	0.70	9.92	128.20	26.87	179.46	300.75
TCGA-AY-6386	36.74	-0.69	49.62	25.83	20.62	11.30	31.34	173.66	36.25	183.28	291.19
TCGA-A6-5665	36.84	0.77	51.40	70.36	100.68	30.67	51.66	119.78	13.60	218.97	124.54
TCGA-D5-6898	37.18	-0.26	44.95	67.13	23.09	25.59	28.82	356.40	74.65	209.26	384.00
TCGA-CM-6162	37.28	1.10	79.29	73.56	82.96	44.62	70.34	762.76	84.30	306.23	629.26
TCGA-G4-6320	37.50	-1.08	56.21	7.86	29.64	7.64	95.65	100.50	15.24	190.98	225.87
TCGA-5M-AAT6	37.76	1.71	99.75	114.84	116.06	16.65	84.00	608.14	83.74	193.41	483.18
TCGA-AY-A69D	37.83	-1.21	62.15	10.65	15.86	9.72	76.18	123.29	13.99	178.28	294.35

`

TCGA-G4-6306	38.06	-0.10	58.66	31.67	40.21	4.40	16.01	81.03	7.60	263.80	128.02
TCGA-4T-AA8H	38.43	-1.20	57.18	13.89	5.85	2.01	17.36	43.14	5.41	204.33	234.65
TCGA-F4-6809	39.11	0.11	102.69	68.64	38.01	4.45	245.07	304.70	68.56	247.23	837.98
TCGA-NH-A6GC	39.25	-0.83	63.69	47.88	19.81	3.34	57.40	129.18	110.52	189.38	269.75
TCGA-A6-6652	40.20	-0.83	48.39	48.27	7.71	2.96	2.41	97.72	6.85	246.51	271.47
TCGA-G5-6572	40.73	0.47	67.15	120.72	31.89	10.50	42.52	450.40	70.18	694.59	274.09
TCGA-CM-6170	40.81	-1.02	66.23	15.38	15.74	14.55	43.61	247.88	33.43	214.30	353.42
TCGA-DM-A1D9	41.86	-1.25	37.58	6.62	13.11	0.91	8.07	104.87	23.71	174.90	210.11
TCGA-AF-A56K	43.69	-0.18	85.02	81.15	31.30	5.64	37.25	299.05	36.70	256.03	683.66
TCGA-CM-5861	45.36	-0.10	33.47	69.26	39.37	13.07	7.24	102.88	13.85	226.24	207.46
TCGA-CM-5863	45.96	-0.33	37.96	72.61	19.99	2.95	31.32	213.77	32.80	295.36	430.14
TCGA-4N-A93T	47.68	-0.78	62.58	46.93	8.29	3.57	51.77	97.59	11.13	117.37	318.63
TCGA-AY-A71X	48.33	-0.71	38.18	65.33	5.22	6.44	11.24	53.42	7.07	185.17	236.95
TCGA-F4-6704	48.67	-0.40	60.02	29.34	36.98	8.64	81.69	392.23	94.72	258.65	753.47
TCGA-DC-6682	50.92	0.37	36.30	110.87	28.27	2.13	116.36	117.37	27.11	138.02	196.72
TCGA-G4-6299	52.07	1.73	87.97	75.98	105.44	6.26	42.60	386.26	40.67	275.19	270.12
TCGA-NH-A8F7	271.86	-1.39	35.74	0.40	0.69	4.94	2.32	45.16	1.94	177.00	184.47

`

Table S3. Oligonucleotides used in this study.

Name	Sequence (5′ to 3′)
45 bp ISD-F	TACAGATCTACTAGTGATCTATGACTGATCTGTACATGATCTACA
45 bp ISD-R	TGTAGATCATGTACAGATCAGTCATAGATCACTAGTAGATCTGTA
Mouse Ifnb-F	TCCGAGCAGAGATCTTCAGGAA
Mouse Ifnb-R	TGCAACCACCACTCATTCTGAG
Mouse GAPDH-F	AGGTCGGTGTGAACGGATTTG
Mouse GAPDH-R	TGTAGACCATGTAGTTGAGGTCA
Huamn IFNb-F	TCCAAATTGCTCTCCTGTTG
Huamn IFNb-F	GCAGTATTCAAGCCTCCCAT
Human GAPDH-F	GAGTCAACGGATTTGGTCGT
Human GAPDH-R	TTGATTTTGGAGGGATCTCG
cGAS KO F1	TATGTACAGGAACCCGTGCAG
cGAS KO R1	CTTAACCACTGAGCCATCTCTAG
cGAS KO F2	TTCACTAAATAGACCAAGCTGCTG
cGAS KO R2	ATGACTCAGCGGATTTCCTCG
Mouse Isg15-F	GGAACGAAAGGGGCCACAGCA
Mouse Isg15-R	CCTCCATGGGCCTTCCCTCGA
Mouse Ifit1-F	TCTAAACAGGGCCTTGCAG
Mouse Ifit1-R	GCAGAGCCCTTTTTGATAATGT
Mouse Ifit3-F	TGAACTGCTCAGCCCACA
Mouse Ifit3-R	TCCCGGTTGACCTCACTC

`

Mouse Nfkibia-F	AAGAAGGAGCGCTTGGTGG
Mouse Nfkibia-R	GTCTCCCTTCACCTGACCAA
Mouse ICAM1-F	TGCTCAGGTATCCATCCATCC
Mouse ICAM1-R	TGCTCAGGTATCCATCCATCC
Mouse SELE-F	CTGCTGGAGTCATGAATGCC
Mouse SELE-R	ACCAGATGTGTGTAGTCCCG
Mouse IL6-F	TTCTTGGGACTGATGCTGGT
Mouse IL6-R	CAAGTGCATCATCGTTGTTCA
Mouse Mip1a-F	CAACCAAGTCTTCTCAGCGC
Mouse Mip1a-R	CTTTGGAGTCAGCGCAGATC
Mouse Cxcl10-F	ATGACGGGCCAGTGAGAATG
Mouse Cxcl10-R	ATTCCGGATTCAGACATCTCT
Mouse Alt-F	ATGGACTGAAGGGGAAGGTG
Mouse Alt-R	AGAAGGTGATGGGTCTCTGC
Mouse Got1-F	CTCCTCCGGTTCTGGTCTTT
Mouse Got1-R	CCCCAAGAACTAGGCGAGAA
Mouse Dhfr-F	ACCATTGAACTGCATCGTCG
Mouse Dhfr-R	CCTCGTGGTGGTTCTTTGAG
Mouse Odc1-F	AAGGACGAGTTTGACTGCCA
Mouse Odc1-R	AGCCACCTCAGATGCTTCTT
Mouse Srm-F	TTCCGAGAGACCTGCAGC
Mouse Srm-R	TGGCGATCATCTCCTGGTAG

`

Mouse Sms-F	CAGCACGCTCGACTTCAAG
Mouse Sms-R	AGCTGCCATTCTTGTTCGTG
Mouse Cs-F	GCTCTACTCACTGCAGCAAC
Mouse Cs-R	CCATGTTGCTGCTTGAAGGT
Mouse Hk2-F	GATCGCCTGCTTATTCACGG
Mouse Hk2-R	TGCTGTAGGGTGTGTGGTAG
Mouse Ldha-F	GTAAGTCCTCAGGCGGCTAC
Mouse Ldha-R	GTAAGTCCTCAGGCGGCTAC
Mouse Pygl-F	GAATGTGGCCGAGCTGAAAA
Mouse Pygl-R	TAGTGCTGCTGTGTACGGAT
Mouse Gys1-F	GTCTCTCTGTGTCCTCGCTT
Mouse Gys1-R	TGGCGATCATCTCCTGGTAG
Mouse Hsl-F	CAAGCAGGGCAAAGAAGGAT
Mouse Hsl-R	CCGAACACCTGCAAAGACAT
Mouse Acc1-F	CATCTCCGTTGGCCAAAACT
Mouse Acc1-R	GCCAAACCATCCTGTAAGCC
KR-A GAS-N (1-160aa)	ATGCAGCCTTGGCACGGAGCAGCCATGCAGGCAGCTTCCGAGGCCGGAGCCACTGCCCCCGCAGCTTCC<br>GCAGCAAATGCCGCCGGCGCCCCGATGGATCCCACCGAGTCTCCGGCTGCCCCCGAGGCCGCCCTGCCT<br>GCAGCGGGAGCATTCGGCCCCGCCGCAGCGTCGGGATCCGCGCAGGCAGCGAGCGCCCCGGACACCCA<br>GGAGGCACCGCCCGTCGCCGCAACTGGGGCCGCGGCCGCAGCCGCCCCTCAGGCAGCCCAGGACACGC<br>AGCCGTCTGACGCCACCAGCGCCCCTGGGGCAGAGGGGCTGGAGCCTCCTGCGGCTGCCGAGCCGGCT<br>CTTTCCGCAGCTGGTTCTTGCGCCCAGGCAGGCGCGGCCTGCTCCACGGCACCAGCACCTCCGCCCGGGC<br>CCTGGGACGTGCCCAGCCCCGGCCTGCCGGTCTCGGCCCCCATTCTCGTAGCCGCAGATGCGGCGCCT

`
